# Improving the early diagnosis and clinical outcomes of shock patients via laser speckle contrast imaging assessment of peripheral hemodynamics

**DOI:** 10.1016/j.isci.2024.111307

**Published:** 2024-11-04

**Authors:** Meng-Che Hsieh, Jin-Jia Hu, Yan-Ren Lin, Shih-Yu Li, Pei-You Hsieh, Congo Tak Shing Ching, Lun-De Liao

**Affiliations:** 1 Institute of Biomedical Engineering and Nanomedicine, National Health Research Institutes, 35, Keyan Road, Zhunan Town, Miaoli County 350, Taiwan; 2 Doctoral Program in Tissue Engineering and Regenerative Medicine, National Chung Hsing University, 145, Xingda Road, South District, Taichung City 402, Taiwan; 3 Department of Mechanical Engineering, National Yang Ming Chiao Tung University, No. 1001, Daxue Rd. East Dist., Hsinchu City 300093, Taiwan; 4 Department of Emergency and Critical Care Medicine, Changhua Christian Hospital, Changhua, Taiwan; 5 Graduate Institute of Biomedical Engineering, National Chung Hsing University, 145, Xingda Road, South District, Taichung City 402, Taiwan; 6 Department of Electrical Engineering, National Chi Nan University, Puli Township 54561, Taiwan; 7 International Doctoral Program in Agriculture, National Chung Hsing University, Taichung 402, Taiwan; 8 Advanced Plant and Food Crop Biotechnology Center, National Chung Hsing University, Taichung 402, Taiwan

**Keywords:** Health sciences, Physics, Optics

## Abstract

Shock is defined as a critical circulatory failure that requires prompt diagnosis to optimize patient outcomes. Traditional diagnostic methods have limitations, including contact-based measurements, high costs, and lengthy procedures. The study evaluated the efficacy of laser speckle contrast imaging (LSCI), a noncontact technique, for assessing peripheral hemodynamics in shock patients. Results showed that LSCI accurately captured dynamic changes in blood flow, revealing early indicators of shock. ROI diff and ROI diff/ROI_2_ values significantly differed between shock patients and healthy controls. Spearman’s correlation analysis revealed associations between ROI diff and key physiological parameters, such as blood pressure and heart rate. ROC analysis revealed that ROI diff and ROI diff/ROI_2_ had strong accuracy (72.5% and 82.5%, respectively) for detecting shock. Additionally, LSCI reduced testing time by over 50%, offering faster assessments. These findings demonstrate the potential of LSCI to improve diagnosis and management of shock, especially in complex clinical environments.

## Highlights

• LSCI reliably detects shock with ROI diff/ROI_2_ showing 82.5% accuracy
• LSCI reduces test time by over 50% compared to traditional methods
• Noncontact LSCI improves shock detection while lowering infection risk
• LSCI demonstrates significant correlation with key physiological parameters

## Graphical abstract

**Figure:**
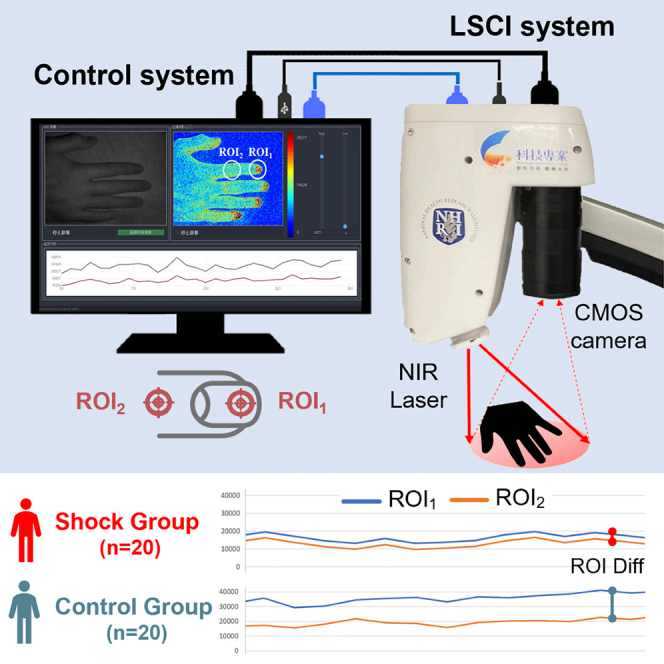

## Introduction

Shock is a critical medical condition characterized by inadequate perfusion of systemic organs and can lead to severe consequences, including organ dysfunction and death. This condition is characterized by impaired cardiovascular blood flow, excessive metabolic consumption, or inadequate oxygen supply to tissues, resulting in cell and tissue hypoxia. As a result, shock can lead to multisystem organ dysfunction, which in turn can culminate in irreversible damage to vital organs and ultimately fatal outcomes.^1^ Shock is a common condition in critical care medicine, affecting approximately one-third of patients in intensive care units (ICUs).^2^ Identifying patients with shock and administering appropriate diagnostic and therapeutic interventions in the early stages are crucial, as these interventions can substantially improve patient prognosis. Delayed diagnosis or failure to initiate treatment promptly can have irreversible consequences. It is crucial for healthcare professionals to determine whether a patient is in a state of shock and to identify the type of shock when the initial signs of abnormality are manifested. During the early stages of shock, patients commonly experience clinical symptoms such as decreased blood pressure, increased lactate concentration, rapid heart rate, and various other manifestations, including pallid skin, cold and clammy extremities, reduced urine output, and possible loss of consciousness.^3^^,^^4^ Shock leads to decreased blood volume, decreased cardiac output, vasodilation, and an abnormal heart rate in patients. These factors can occur independently or coexist, and depending on the specific combination of factors, they can lead to different types of shock.^5^ Despite the diverse natures of shock, a common characteristic of all types of shock is the early onset of hypotension. Detecting and analyzing the clinical indicators of shock can be a useful way to diagnose this condition early and provide timely treatment. By doing so, healthcare professionals can effectively manage the condition, improving patient outcomes.

Shock occurs due to a sudden decrease in blood volume, which leads to tissue hypoxia due to a significant loss of red blood cells.^6^ Peripheral blood circulation has substantial clinical value in the diagnosis and treatment of shock.^7^ Shock is a form of circulatory dysfunction that is caused by the body’s inability to maintain sufficient perfusion pressure, resulting in ischemia and hypoxia in various tissues and organs. Peripheral blood circulation refers to blood flow between tissues and cells in the most distant regions of the body, including the fingers, toes, and skin. When shock occurs, peripheral blood circulation is typically the first to be affected, as the body prioritizes adjustments to protect vital organs, such as the heart and brain, resulting in decreased peripheral blood perfusion.^8^^,^^9^ In shock patients, abnormalities in the peripheral blood circulation often manifest as decreased blood pressure, cold and cyanotic skin, and a weak or difficult-to-detect pulse.^10^ Therefore, monitoring the state of peripheral blood circulation is crucial for the early identification of shock.

Various methods and instruments are used in clinical practice to determine whether a patient is experiencing shock. Common indicators include the capillary refill time (CRT), the shock index (SI), arterial lactate levels, and blood pressure. In emergency medical situations, the CRT is often used to assess shock status. This involves pressing a patient’s fingertip to turn it white, maintaining this pressure for 15 s, and then releasing this pressure and measuring the time required for the fingertip to return to its full pink color. In healthy subjects, this recovery time is typically less than 2.5 s. This method is a quick and simple way to assess shock and is frequently used in ambulances. However, the use of the CRT in clinical practice has limitations because of subjective differences in observations and the inability to fully quantify the results.^11^^,^^12^ In the emergency room, the SI, lactate levels, and blood pressure are commonly used indicators to assess shock. The lactate level is a biochemical marker that is often used in the diagnosis of shock, and it reflects anaerobic metabolism due to inadequate circulatory oxygen delivery. The SI is calculated by dividing the heart rate (HR) by the systolic blood pressure (SBP). An SI greater than 0.9 often indicates a high probability of shock.^13^^,^^14^ Furthermore, in clinical settings, the mean arterial pressure (MAP) and SBP are common indications for observing shock. The equipment that is commonly used to assist in determining whether a patient is in shock includes noninvasive sphygmomanometers, arterial catheters, or ultrasound cardiac output monitors (USCOMs), which are commonly used to measure blood pressure, but all three of these devices carry the risk of endangering a patient who is in shock. Noninvasive sphygmomanometers are useful; however, using these instruments takes time, and their use cannot be continuously tracked. Although arterial catheters offer precise and ongoing data, this approach is invasive and requires longer durations, which can result in additional difficulties.^15^ The USCOM device offers absolute accuracy in blood pressure measurement, and it can be used relatively rapidly; however, its high cost makes it inaccessible to all front-line healthcare professionals who treat shock patients. In the context of developing technologies, blood biomarker measurement methods and imaging scans (CT, MRI, and ultrasound) all have benefits and limitations in terms of the speed of diagnosis as well as the precision and working mechanism of the tests. For example, CT and MRI provide detailed imaging information but are expensive, require specialized skills, and have slower diagnostic speeds.^16^ Ultrasound technology, while fast and noninvasive, demands high technical proficiency of the operator, and the results can be influenced by operator variability. Nevertheless, traditional approaches that are used in the early identification and real-time monitoring of shock have numerous limitations, including the absence of noninvasive examinations, the necessity of professional intervention, and slow detection rates. Additionally, there are challenges in the practical clinical application of emerging technologies, including high equipment costs, operational complexity, and patient discomfort. The crucial limitation of all three devices is that their use requires direct contact with the patient. In cases where patients have open wounds or extensive trauma, as seen in events such as the 2012 Taiwan Formosa Fun Coast dust explosion, which left numerous patients with extensive burns, healthcare personnel may face challenges in diagnosis.^17^ In future potential warfare scenarios, the number of casualties may suddenly increase to hundreds or thousands. Patients experiencing shock due to explosive impacts must receive immediate treatment due to the severity of their injuries. However, for individuals who cannot be examined by healthcare providers via contact-based diagnostics, the critical treatment window could ultimately be missed. Thus, for a number of conditions, the use of a quick and contactless shock detector is needed, and laser speckle contrast imaging (LSCI), which is used in this study, provides both benefits.

The system architecture of LSCI involves the use of a laser that emits single or multiple wavelengths of light. The laser light is diffused through a diffuser to illuminate the test subject uniformly, resulting in random scattering. After passing through a lens that filters specific wavelengths, the scattered light enters the camera and generates a speckle pattern image. Subsequently, computer processing is used to calculate the speckle contrast, thereby generating an image for blood flow visualization.^18^^,^^19^ Recent advances in LSCI technology have significantly increased its diagnostic capabilities and clinical applications. Innovations in image processing algorithms and the integration of LSCI with other imaging modalities have improved spatial and temporal resolution, data quality, and measurement precision, expanding its clinical utility. LSCI is a dynamic light scattering (DLS)-based spin-off technique that is widely used for noninvasive imaging of blood flow in the brain, skin, muscles, and other biological tissues. These techniques, including laser Doppler flowmetry (LDF), diffusing wave spectroscopy (DWS), and Doppler optical coherence tomography (DOCT), leverage the principles of DLS to analyze fluctuations in intensity caused by the motion of scattering particles. Due to recent advances in DLS-based imaging, these technologies show great promise in applications such as brain blood flow monitoring, skin perfusion measurements, and noninvasive blood microcirculation characterization.^20^ A recent study by Sdobnov et al.2024^21^ highlighted the application of LSCI in visualizing cerebral hemodynamics in mice, particularly during and after cardiac arrest, emphasizing the potential of LSCI in microcirculation research because of its high temporal resolution and noninvasiveness. Similarly, Zherebtsov et al.^22^ demonstrated the clinical utility of DLS sensors in detecting microvascular changes associated with aging and diabetes, validating the potential of LSCI technology for noncontact diagnostics. Zharkikh et al.^23^ reviewed biophotonics technologies, including LSCI, for detecting diabetic complications, placing LSCI within a broader context of noninvasive diagnostic applications. Studies by Kalchenko et al.^24^^,^^25^ further advanced the field by addressing the limitations of LSCI and exploring its brain imaging applications, providing essential insights into the differentiation of vessel functionality. Additionally, the special issue on “Dynamic Light Scattering in Biomedical Applications”^26^ compiled the latest breakthroughs and future directions for DLS-based technologies, further solidifying the potential of LSCI for widespread clinical integration. Previous studies have indicated that when LSCI is used, neurovascular blood flow can be detected in real time, and this approach has been tested in dynamic intraoperative situations.^27^ Furthermore, continuous blood flow visualization during neurosurgery has been achieved via LSCI, providing valuable real-time feedback to surgeons.^28^ Preclinical studies have also validated the use of LSCI for real-time visualization of cerebral blood flow during cerebrovascular surgery, highlighting its practical applications in critical surgical procedures. Currently, in clinical medicine, LSCI is used in ophthalmology to record variations in retinal blood flow, in dermatology to assess the depth of burns in patients and to observe facial microvasculature, and in neurosurgery and gastrointestinal surgery as an additional tool for surgery and diagnostics.^29^^,^^30^ These studies and clinical applications underscore the versatility of LSCI, demonstrating its emerging role in real-time surgical feedback as well as its potential for broader integration across various medical fields.

LSCI is a noncontact, medical imaging method that allows rapid visualization of blood perfusion. LSCI has widespread use in clinical settings. However, LSCI has not been used to observe peripheral blood circulation to identify patients in shock. Unlike contact-based devices, LSCI captures speckle patterns via a high-resolution camera after a biological tissue is illuminated with laser light. Therefore, LSCI does not require any physical contact with the patient. LSCI simply needs to be aimed at the region of interest (ROI) for imaging, substantially reducing the risk of infection. With the use of LSCI technology, healthcare professionals can immediately observe blood flow within the scanning area. After the initial blood flow image is obtained, two distinct regions of interest (ROIs_1_ and ROI_2_) can be selected on the screen for tracking analysis. By calculating and comparing the speckle contrast in these two areas, the difference in blood flow velocity can be determined. Subtracting the speckle contrast of these two regions yields the ROI difference (ROI diff), which clearly reflects the difference in blood flow between these intervals. In shock patients, the body responds to symptoms of low blood pressure caused by shock by activating the cardiovascular system directly through the medulla oblongata and reticular activating substances in the brainstem. This cardiovascular activation occurs through the sympathetic nervous system, increasing heart rate, myocardial contractility, and peripheral blood vessel constriction.^31^^,^^32^ When peripheral blood vessels constrict in the human body, the flow of blood entering the constricted vessels is substantially reduced to increase blood pressure to a normal state. To assess the effectiveness of LSCI in detecting peripheral blood circulation and identifying patients who are in shock, this study was conducted in partnership with Changhua Christian Hospital. The aim of this study was to evaluate the accuracy and practicality of LSCI in identifying shock patients in a clinical setting.

## Results

This section presents findings from our two-phase study on the use of LSCI to diagnose shock. The main results focus on our primary investigation (Phase 1), which aimed to evaluate the performance of LSCI in identifying shock patients and establishing diagnostic criteria. To comprehensively assess the clinical value of LSCI, we analyzed common shock detection standards and their clinical application, and then, we conducted in-depth comparisons between LSCI and traditional methods. We then present findings from our supplementary study (Phase 2), which explored the performance of LCSI across different shock types and its relationship with heart rate variability (HRV) in shock patients. By presenting both phases and evaluating common shock detection standards, we provide a thorough assessment of LSCI as a novel shock diagnosis tool, address its standalone performance and compare it to established clinical standards. This approach offers insights into the potential value and applications of LCSI in clinical practice.

### Laboratory safety for participants and medical personnel

No safety-related incidents occurred during either phase of this study, in which medical personnel used LSCI to diagnose shock. In both phases, patients in the experimental groups were experiencing shock or were about to enter that state. The experimental groups consisted of 20 shock patients each, for a total of 40 shock patients across the two phases. Healthcare providers closely monitored vital signs using various instruments to ensure patient safety while using LSCI to assess shock symptoms. To prevent laser-induced injuries, the intensity of the laser used in the clinical trials was set to a safe level (600 mA), thus posing no threat to users. In both experimental groups, shock participants, including some elderly individuals, were brought to the hospital under emergency conditions. As shown in Tables 1 and S1, most patients had at least one condition in their past medical history (PMH), including various chronic conditions such as hypertension (H.T.N.), diabetes mellitus (D.M.), or cardiovascular disease (C.V.D.). Due to these varied medical histories, medical personnel closely monitored the volunteers' physiological states, especially those of older individuals, to prevent accidental injury during testing. The examination of shock patients was facilitated by the adjustable position and noncontact nature of LSCI. Healthcare professionals could easily adjust the LSCI position, allowing measurements to be taken even when patients were immobile. This high maneuverability and noncontact design allowed for shock assessments without causing additional harm to elderly or severely injured patients, thus ensuring a safe and effective testing environment in both phases of the study. In the second phase, additional safety measures were implemented for the simultaneous ECG measurements, ensuring that the combined use of LSCI and ECG posed no additional risks to the patients.

**Table 1:** Basic information of the study group and the control group

		Study Group		Control Group		*p*-value	Cohen’s *d*
		*N* = 20		*N* = 20			
		n(%); Mean ± SD	Median	n(%); Mean ± SD	Median		
Gender	Male	14 (70)	–	8 (40)	–	–	–
	Female	6 (30)	–	12 (60)	–	–	–
Age (year)	–	68.8 ± 15.4	68.0	41.7 ± 12.0	39.5	<0.001	−2.0
Height (cm)	–	160.5 ± 8.5	162.5	164.2 ± 6.7	163.5	–	0.5
Weight (kg)	–	55.2 ± 8.9	55.0	66.9 ± 13.3	64.0	<0.05	1.0
BP (mmHg)	MAP	71.4 ± 18.5	69.2	93.8 ± 12.9	91.7	–	1.4
	SBP	91.9 ± 18.6	93.5	127.4 ± 19.4	126.0	<0.001	1.9
	DBP	61.3 ± 19.1	58.5	77.0 ± 11.1	78.0	<0.05	1.0
BT (°C)	–	36.8 ± 1.5	36.5	36.2 ± 0.1	36.2	–	−0.6
HR (bpm)	–	112.9 ± 24.4	111.0	76.9 ± 7.7	78.0	<0.001	−2.0
RR (breathing/min)	–	21.9 ± 3.2	21.0	18.7 ± 0.9	18.0	<0.001	−1.4
SpO2 (%)	–	94.6 ± 6.9	97.5	99.2 ± 0.5	99.0	<0.05	0.9
SI (HR/SBP)	–	1.3 ± 0.4	1.2	0.6 ± 0.1	0.6	<0.001	−2.5
Lactate (mmol/L)	–	4.5 ± 3.8	3.2	–	–	–	–
ROI1	–	33674.6 ± 15748.0	31481.1	36319.7 ± 11362.2	36285.7	–	0.2
ROI2	–	24656.0 ± 11762.1	20375.7	21681.6 ± 7396.1	20284.5	–	−0.3
ROI Difference	–	9018.7 ± 7620.0	6249.0	14638.1 ± 5970.7	14406.0	<0.05	0.8
Medical History	H.T.N.	8 (40)		1 (5)		–	–
	D.M.	9 (45)		2 (10)		–	–
	H.L.D.	3 (15)		0		–	–
	C.V.D.	6 (30)		0		–	–
	L.C.	1 (5)		0		–	–
	C.V.A.	2 (10)		0		–	–
	R.F.	2 (10)		0		–	–
	GI diseases	5 (25)		1 (5)		–	–
	I.T.P.	2 (10)		0		–	–
	C.A.	8 (40)		5 (25)		–	–

Hypertension (H.T.N.), diabetes mellitus (D.M.), hyperlipidemia (H.L.D.), cardiovascular disease (C.V.D.), liver cirrhosis (L.C.), cerebral vascular accidents (C.V.A.), renal failure (R.F.), gastrointestinal diseases (GI disease), immune thrombocytopenia purpura (I.T.P.), and cancer (C.A.).

### Differences in the physiological data of the enrolled subjects

This study included two phases of clinical research conducted at Changhua Christian Hospital. The first phase (IRB 211116) included 20 shock patients (experimental group) and 20 healthy subjects (control group). The second phase (IRB 230705) also included 20 shock patients and 20 healthy subjects, thus yielding a total sample of 80 participants across both phases. As shown in Table 1 for the first phase and Table S1 for the second phase, both studies presented significant differences (*p* < 0.05) in physiological parameters between the experimental and control groups, including systolic blood pressure (SBP), diastolic blood pressure (DBP), mean arterial pressure (MAP), heart rate (HR), respiratory rate (RR), oxygen saturation (SpO_2_), and shock index (SI). These differences highlight the notable variations in physiological values between shock patients and healthy subjects across both phases of the study. Furthermore, in both phases, the *p* value of the difference in the ROI diff values between the experimental and control groups was less than 0.05 (0.006 in the first phase), indicating significant differences. This finding suggests a distinct difference in peripheral blood flow between shock patients and healthy subjects, which was consistently observed in both phases of the study. In the second phase, additional analysis of heart rate variability (HRV) parameters was conducted, further distinguishing the physiological differences between shock patients and healthy subjects. These results are detailed in Table S2. These consistent findings across both phases of the study strengthen the reliability of LSCI for detecting physiological differences between shock patients and healthy individuals.

### Comparison of physiological data between the experimental and control groups

**Figure 1:**
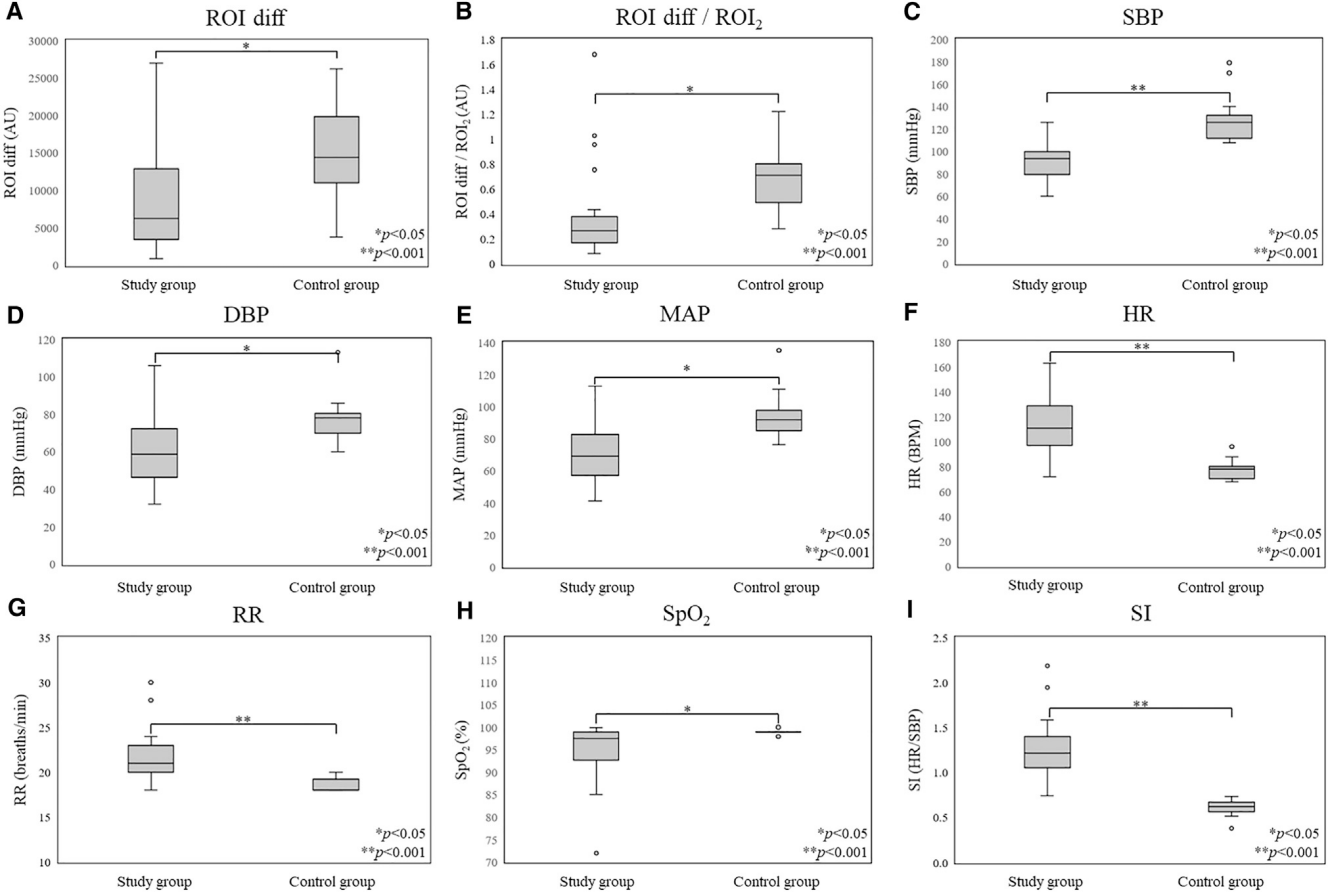
Box and whisker plots of physiological data from the study group compared with those from the control group and *p* values (A) The *p* value of ROI diff was less than 0.05, indicating a significant difference; however, the box-and-whisker plots show that the data ranges of the two groups overlapped, which means that the data of the study group and the control group were too similar to each other. (B) A *p* value <0.05 for ROI diff/ROI_2_ was considered to indicate statistical significance; from the graph, the overlap between the two sets of data was much lower than that of the ROI, which means that the overlap of the data was quite low. (C) A *p* value for SBP <0.05 indicated a significant difference; the median and mean values in the study group were 93.5 mmHg and 91.85 mmHg, respectively, whereas those in the control group were approximately 127 mmHg; the values in the study group were obviously lower than those in the control group. (D) A *p* value <0.05 for DBP was considered to indicate a significant difference; the median and mean values in the study group were 58.5 mmHg and 61.3 mmHg, respectively, whereas those in the control group were 78 mmHg and 77 mmHg, respectively; the values in the study group were lower than those in the control group. (E) A *p* value <0.05 for the MAP was considered significant, and the median arterial pressure was significantly greater in the control group than in the study group. (F) A *p* value <0.05 for HR was considered to indicate a significant difference; the minimum value in the study group was only slightly greater than the maximum value in the control group, and the HR in the study group was greater than that in the control group. (G) A *p* value <0.05 for the RR was considered to indicate a significant difference; the minimum value in the study group was equal to the median value in the control group, 18 breaths/min, indicating that the experimental group had more data than did the control group. (H) A *p* value <0.05 for SpO_2_ was considered to indicate statistical significance; although the study group exhibited a wider range of oxygen concentrations, the values in the control group were closer to the ideal range of 99%–100%, whereas those in the study group were in the lower end of the range. (I) A significant difference was indicated for a *p* value of SI < 0.05; the values of the data in the study group were significantly greater than those in the control group, and there was essentially no overlap in the interquartile range between the two groups. These findings are consistent with the conclusion that the SI values of shock patients are almost all greater than 0.9.

In the first phase of our study (IRB 211116), the Mann‒Whitney U test was used to compare the physiological data between the experimental and control groups, as well as the ROI diff value, which was used for shock identification, to verify whether the *p* values supported the alternative hypothesis. Figure 1A shows that the *p* value of the difference in the ROI diff values between the experimental and control groups was less than 0.05, indicating a significant difference. However, the gray shaded area indicates a potential overlap in the data, which could lead to misdiagnoses in shock identification. In Figure 1B, using ROI_2_ as a reference point, the difference between the ROI_1_ and ROI_2_ values was calculated and divided by the ROI_2_ value to obtain the ROI diff/ROI_2_ value, which was used for further assessment of the shock patients. The *p* value of the difference in the ROI diff/ROI_2_ values between the two groups was less than 0.05, indicating a significant difference. In addition, the boxplots did not reveal data overlap, confirming a lower misdiagnosis rate in shock identification. In terms of SBP (Figure 1C), the mean values for the experimental group and the control group were 91.85 mmHg and approximately 127 mmHg, respectively. The values of the control group were nearly 40% greater than those of the experimental group, and the *p* value was less than 0.05, indicating a significant difference. For DBP (Figure 1D), similar to SBP, the average value in the experimental group was 61.3 mmHg, whereas the average value in the control group was 77 mmHg. The experimental group exhibited a decrease of 26%, and the *p* values for differences in SBP and MAP between the two groups were less than 0.05, indicating significant differences. Figure 1E shows that the *p* value of the difference in the MAP between the two groups was <0.05, indicating a significant difference, with the median arterial pressure in the control group being significantly greater than that in the experimental group. Figure 1H shows that the *p* value of the difference in the SpO_2_ values between the two groups was <0.05, indicating a significant difference. Although the experimental group exhibited a wider range of oxygen saturation, the oxygen saturation was closer to the ideal range of 99%–100%. In contrast, the experimental group presented values that fell within a worse range, which is indicative of clinical signs that can occur in shock patients. Figures 1F and 1G show that the overall HR and RR in the experimental group were significantly higher than those in the control group, with *p* values less than 0.05. On the basis of these observations, the clinical performance in the experimental group indeed aligned with the characteristics of shock.

**Figure 2:**
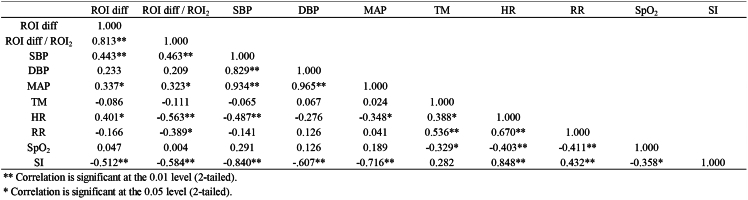
Spearman’s correlation analysis of the LSCI test results with physiological values The correlation between the ROI diff and ROI diff/ROI_2_ values that were detected with LSCI and the physiological values of the participants can be observed in the graphs. The correlation coefficients (*r*_*s*_) of SBP and MAP with the ROI diff value and the physiological values of SBP and MAP were greater than 0.3, indicating a significant positive correlation. The *r*_*s*_ of the HR was less than −0.4, indicating a significant negative correlation. The clinical performance of SBP, MAP, and HR in shock patients was correlated with the ROI diff value detected by LSCI. Notably, the ROI diff/ROI_2_ value had a greater correlation with SBP and a significant negative correlation with RR and HR. In particular, there was a significant correlation between two-tailed values of *p* < 0.05 for MAP and *p* < 0.01 for SBP, HR, and RR. These findings revealed a greater correlation between the ROI diff/ROI_2_ value and the physiological values of the participants than between the ROI diff value and the physiological values, highlighting the improved accuracy and relevance of the ROI diff/ROI_2_ value in clinical assessments.

Spearman’s correlation coefficient (denoted as *r*_*s*_) was used to assess the correlation among ROI diff values, ROI diff/ROI_2_ values, and the physiological values that were used to identify shock patients. Patients with shock typically exhibit clinical features such as decreased SBP, MAP, and HR. Figure 1A shows that as the ROI diff decreases, the likelihood of being diagnosed with shock increases. According to the data in Figure 2, the *r*_*s*_ value of the correlation of ROI diff with SBP was 0.443 (positive correlation), the *r*_*s*_ value of the correlation of ROI diff with MAP was 0.337 (positive correlation), and the *r*_*s*_ value of the correlation of ROI diff with HR was 0.401 (positive correlation). These results indicate a certain level of positive correlation between the ROI diff values and these three physiological values. The correlation analysis for the ROI diff/ROI_2_ values revealed a similar trend, with *r*_*s*_ values of 0.463 between ROI diff/ROI_2_ and SBP, 0.323 between ROI diff/ROI_2_ and MAP, −0.548 between ROI diff/ROI_2_ and HR, and −0.389 between ROI diff/ROI_2_ and RR. Interestingly, there was a stronger association of ROI diff/ROI_2_ with SBP, HR, and RR. On the other hand, Figure 2 suggests that there is a comparatively lower association between physiological data, including DBP, BT, and SpO_2_, and the ROI diff and ROI diff/ROI_2_ values that were obtained with LSCI.

### Enhanced shock detection using the ROI diff and ROI diff/ROI_2_ values

**Figure 3:**
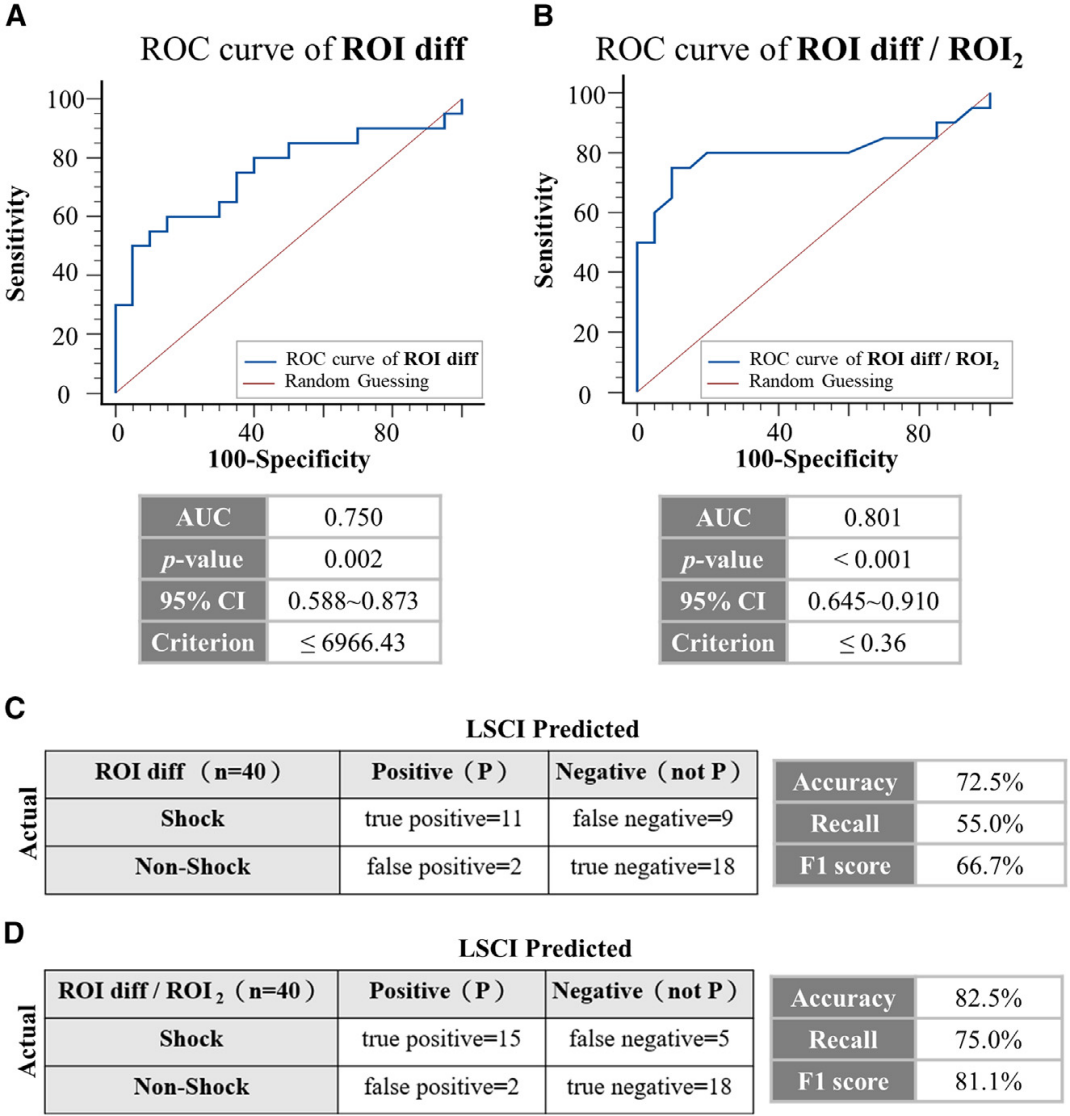
Correlation of the accuracy of the ROI diff and ROI diff/ROI_2_ values (A) ROC curve of the ROI diff value; when the ROC curve was applied to evaluate the ROI diff value, the area under the curve (AUC) was 0.75, and according to the interpretation of the AUC judgment metrics, a range of areas between 0.7 and 0.9 indicated moderate accuracy.^33^ The AUC of the test results reached 0.75, which is greater than the test standard; furthermore, the associated *p* value was less than 0.05, which is a significant difference, suggesting that the use of the ROI diff value as a measurement index has a significant effect. When the ROI diff value was 6966.43, there was an associated criterion that served as a benchmark for diagnosis according to the ROI diff value. (B) ROC curve of ROI diff/ROI_2_; when the ROI diff/ROI_2_ value was analyzed, an AUC value of 0.801 was obtained, which exceeded the AUC value obtained when the ROI diff value was used for analysis. In addition, the associated *p* value was less than 0.001, indicating that the difference was extremely significant, whereas the baseline for diagnosis with the ROI diff/ROI_2_ value was 0.36. (C and D) The ROI diff and ROI diff/ROI_2_ values were used as confusion matrices to identify shock indicators. The associated criterion obtained from the ROC curve was used as a benchmark value for determining shock patients; participants below the benchmark value were determined to be shock patients (positive), and those above the benchmark value were determined to be non-shock patients (negative). The 20 shock patients and 20 healthy subjects were classified according to the baseline values, and then the accuracy, recall, and F1 score were calculated according to the number of people classified. The calculated odds ratios show that the use of the ROI diff/ROI_2_ value as a basis for the identification of shock patients by LSCI improves the overall confidence and accuracy compared with the use of the ROI diff value.

When the ROC curve was applied to evaluate ROI diff (Figure 3A), an observed area under the curve (AUC) of 0.75 was obtained; an AUC ranging from 0.7 to 0.9 indicated moderate accuracy. The AUC of 0.75 for our detection results exceeded the standard for accuracy. Furthermore, the associated *p* value was less than 0.05, indicating a significant difference and suggesting that the ROI diff serves as an effective measurement indicator. An associated criterion was identified when the AUC of ROI diff was 6966.43; this served as the threshold for the use of ROI diff in evaluations. When ROC curve analysis was applied to ROI diff/ROI_2_, the obtained AUC value reached 0.801, surpassing the AUC value obtained when ROI diff was used for detection. Furthermore, the associated *p* value was less than 0.001, indicating extremely significant differences, and the cutoff point for ROI diff/ROI_2_ was 0.36. Through a comparison of the AUC values and *p* values between these two methods, it can be concluded that using ROI diff/ROI_2_ as an indicator for shock detection provides a better basis for assessment and greater accuracy than using ROI diff alone.

The confusion matrices for ROI diff and ROI diff/ROI_2_ were subsequently computed via the associated criterion derived from ROC curve analysis to distinguish between shock patients and non-shock patients. The credibility of the use of LSCI for shock detection was analyzed. Patients who fell below the standard value were classified as positive (P), indicating that they were experiencing shock, whereas those above the standard were considered negative (not P), indicating that they were not experiencing shock. The accuracy, recall, and F1 score for ROI diff and ROI diff/ROI_2_ were calculated on the basis of the actual number of shock and non-shock patients whose clinical data were recorded at Changhua Christian Hospital. Figure 3C shows that ROI diff achieved an accuracy of 72.5%, a recall of 55.0%, and a harmonized F1 score of 66.7% in detecting shock patients. Although ROI diff is highly accurate in predicting patients who are experiencing shock, the correct identification rate of real shock patients among those who are diagnosed as positive falls below 60%. In the end, the combined F1 score of ROI diff did not exceed 70%. This finding indicates that using ROI diff for shock identification in a clinical setting may pose relative risks. Figure 3D shows that ROI diff/ROI_2_ has an overall F1 score of 81.1%, an accuracy of 82.5%, and a recall of 75.0% with respect to shock detection reliability. It is clear from the statistical analysis that the ROI diff/ROI_2_ value not only achieves an F1 score above 80% by combining the analysis of both indicators but also demonstrates an accuracy above 80% in identifying positive cases and accurately identifying shock patients among those with a positive diagnosis. On the basis of this conclusion, the ROI diff/ROI_2_ value represents a very credible and accurate tool for identifying shock patients.

### Comparison of the ROI diff and ROI diff/ROI_2_ values for identifying shock in different time windows

**Figure 4:**
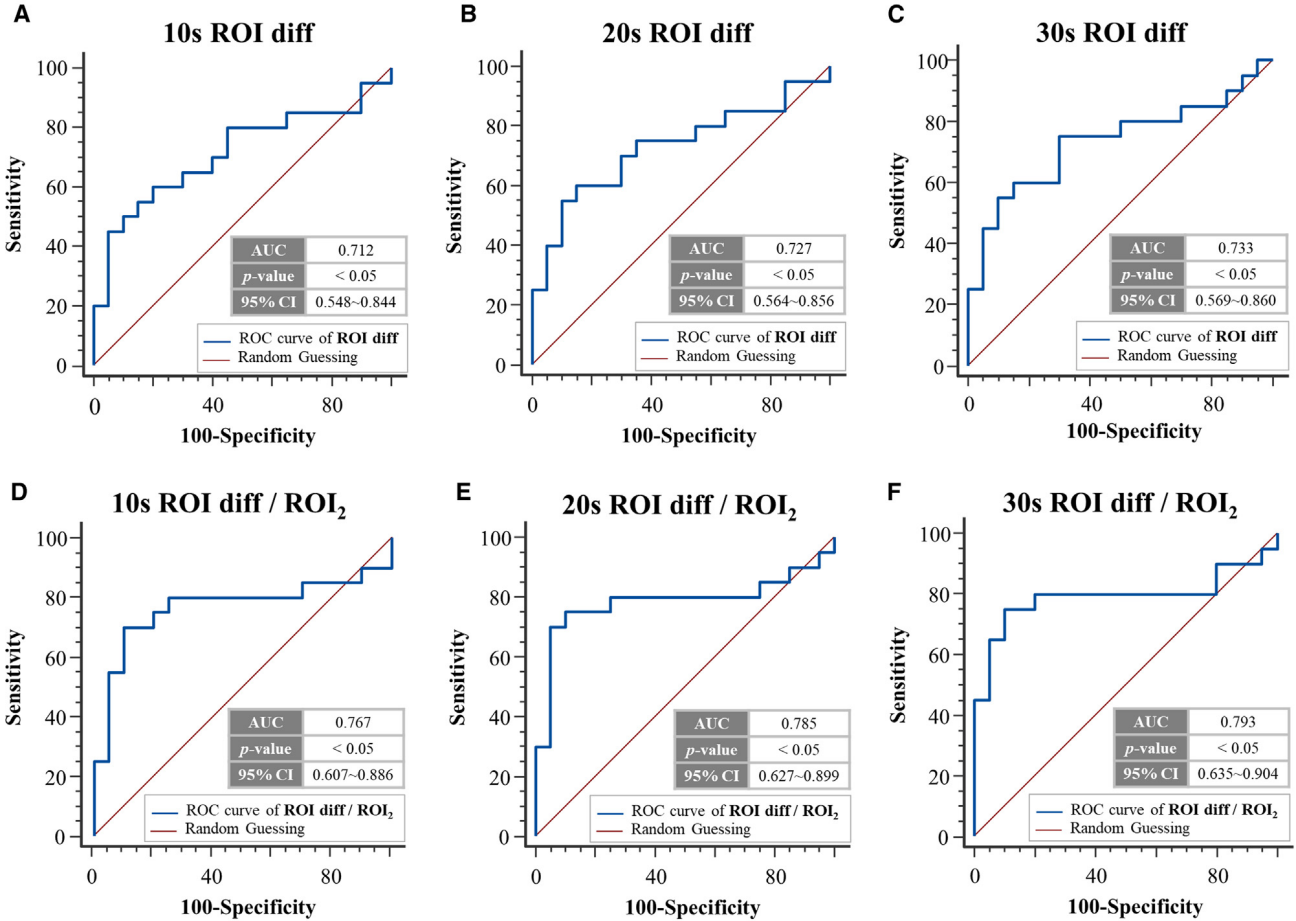
ROC curve analysis for ROI diff and ROI diff/ROI_2_ over different periods (A) ROC curve of the 10-s ROI diff; this graph shows the accuracy of the 10-s ROI diff as a shock determination tool. The horizontal axis represents the specificity, and the vertical axis represents the sensitivity. The AUC was 0.712, and its *p* value was <0.05, indicating statistical significance; the accuracy was 67.5%, and the 95% confidence interval ranged from 0.548–0.844. (B) ROC curve of the 20-s ROI diff; the AUC of the ROI diff at 20-s was 0.727, with statistical significance (*p* < 0.05); the accuracy was 70%, and the 95% confidence interval ranged from 0.564–0.856. (C) ROC curve of the 30-s ROI diff; the AUC for the 30-s ROI diff was 0.733, with statistical significance (*p* < 0.05); the accuracy was 70%, and the 95% confidence interval ranged from 0.569–0.860. (D) ROC curve of the 10-s ROI diff/ROI_2_; the AUC for ROI diff/ROI_2_ at 10 s was 0.569, with statistical significance (*p* < 0.05); the accuracy was 77.5%, and the 95% confidence interval ranged from 0.607–0.886. (E) ROC curve of the 20-s ROI diff/ROI_2_; the AUC for ROI diff/ROI_2_ at 20 s was 0.785, with statistical significance (*p* < 0.05); the accuracy was 80%, and the 95% confidence interval ranged from 0.627–0.899. (F) ROC curve of 30-s ROI diff/ROI_2_; the AUC for ROI diff/ROI_2_ at 30 s was 0.793, with statistical significance (*p* < 0.05); the accuracy was 80%, and the 95% confidence interval ranged from 0.635–0.904.

We conducted a study to investigate the effectiveness of LSCI in identifying shock patients in real time. In this study, we evaluated two indicators (ROI diff and ROI diff/ROI_2_) to identify patients with shock. To obtain a comprehensive understanding of these indicators, we analyzed the same set of samples with time windows set to 10 s, 20 s, and 30 s. This approach allowed us to compare the performance of these indicators at different time scales and observe the effects of time on the sensitivity and specificity in identifying shock patients. First, we analyzed the ROC curves of ROI diff and ROI diff/ROI_2_ in a 10-s time window. As shown in Figure 4, within this time frame, the AUC and accuracy of ROI diff were 0.712 and 67.5%, respectively, and those of ROI diff/ROI_2_ were 0.767 and 77.5%, respectively. Both had *p* values less than 0.05, indicating significant differences. With respect to the 20-s time window, the AUC and accuracy of ROI diff were 0.727 and 70%, respectively, whereas the AUC and accuracy of ROI diff/ROI_2_ were 0.785 and 80%, respectively. Again, both *p* values were less than 0.05, indicating significant differences. In the final analysis within the 30-s time window, the AUC of ROI diff was 0.733, with an accuracy of 70%. For ROI diff/ROI_2_, the AUC was 0.793, with an accuracy of 80%. In both cases, the *p* values were less than 0.05, indicating a significant difference.

The analysis across these three different time windows demonstrates that even in short monitoring periods, the accuracy is consistently greater than 0.7. This finding suggests statistical significance in identifying patients at risk of shock, highlighting the reliability of the measurements. These results not only confirm the feasibility of the ROI diff/ROI_2_ composite indicator but also highlight the stability and high accuracy of LSCI in short-term measurements.

### Evaluation of common shock detection standards

To assess the performance of LSCI for diagnosing shock and to compare this imaging approach with established clinical methods, we analyzed four parameters commonly used in shock detection: MAP, SBP, HR, and SpO_2_. The ROC curves and associated metrics for these parameters are illustrated in Figure S1. Analysis of the ROC curve for MAP (Figure S1A) yielded an AUC value of 0.833, with an accuracy of 85% and a recall of 70% (*p* < 0.0001). The ROC curve for SBP (Figure S1B) yielded an AUC of 0.929, with an accuracy of 90% and a recall of 80% (*p* < 0.01). Analysis of the ROC curve for HR (Figure S1C) yielded an AUC of 0.947, with an accuracy of 90% and a recall of 90% (*p* < 0.001). Analysis of the ROC curve for SpO_2_ (Figure S1D), the AUC was 0.776, with an accuracy of 80% and a recall of 95% (*p* < 0.001). These findings confirm the reliability of MAP, SBP, HR, and SpO_2_ for diagnosing shock, with each parameter significantly discriminating between shock and non-shock states. Among these parameters, HR and SBP exhibited the highest accuracy and reliability.

### Clinical application of shock classification standards

We applied two commonly used shock diagnosis standards from Changhua Christian Hospital to classify patients. The classification criteria were SBP <95 mmHg or MAP <65 mmHg. The results are presented as confusion matrices. Using MAP as a shock predictor (Figure S2A), the model correctly identified 8 shock patients (true positives) and 20 non-shock patients (true negatives) out of 40 patients. However, it missed 12 shock patients (false negatives). It did not misidentify any non-shock patients as having shock (false positives). This resulted in an accuracy of 70.00%, a recall of 40.00%, and an F1 score of 67.14%. Using SBP as the predictor (Figure S2B), the model correctly identified 11 shock patients (true positives) and 20 non-shock patients (true negatives). It missed 9 shock patients (false negatives). It did not misidentify any non-shock patients as having shock (false positives). This results in an accuracy of 77.50%, a recall of 55.00%, and an F1 score of 70.97%. A comparison of the two methods indicated that SBP yielded higher accuracy, recall, and F1 scores than MAP.

### LSCI observations of different types of shock in the second phase study

In the second phase of our research (IRB 230705), we conducted a more detailed analysis of the performance of LSCI across different types of shock. Basic demographic data of the subjects were compared via descriptive analyses and nonparametric tests (Mann‒Whitney U test) (Table S1). The table revealed that the major type of shock in the study group was septic shock (50%), followed by hypovolemic shock (30%), cardiogenic shock (15%), and neurogenic shock (5%). The characteristics of the study and control groups, such as gender, age, height, weight, body temperature, and blood oxygen levels, were not significantly different from each other. However, compared with the control group, the study group presented with significantly lower blood pressure. Additionally, the study group had a significantly higher HR, RR, and CRT as well as a higher prevalence of conditions such as heart disease and cancer. In this second phase, we observed that the mean ROI diff of the study group was significantly lower than that of the control group (*p* < 0.05).

### HRV performance in shock

In the second phase, we analyzed HRV in shock patients. The results shown in Table S2 indicate significant differences in various HRV metrics between the study and control groups. The study group exhibited greater variability and complexity in heart rate dynamics, as evidenced by measures such as the SDRR, RMSSD, PNN50, SD1, and SD2. Additionally, significant differences in frequency domain measures such as VLF and LF power were observed. Nonlinear measures, including SampEn and Higuchi’s fractal dimension, were also different between the groups.

## Discussion

### The effectiveness of LSCI in identifying patients with shock

**Figure 5:**
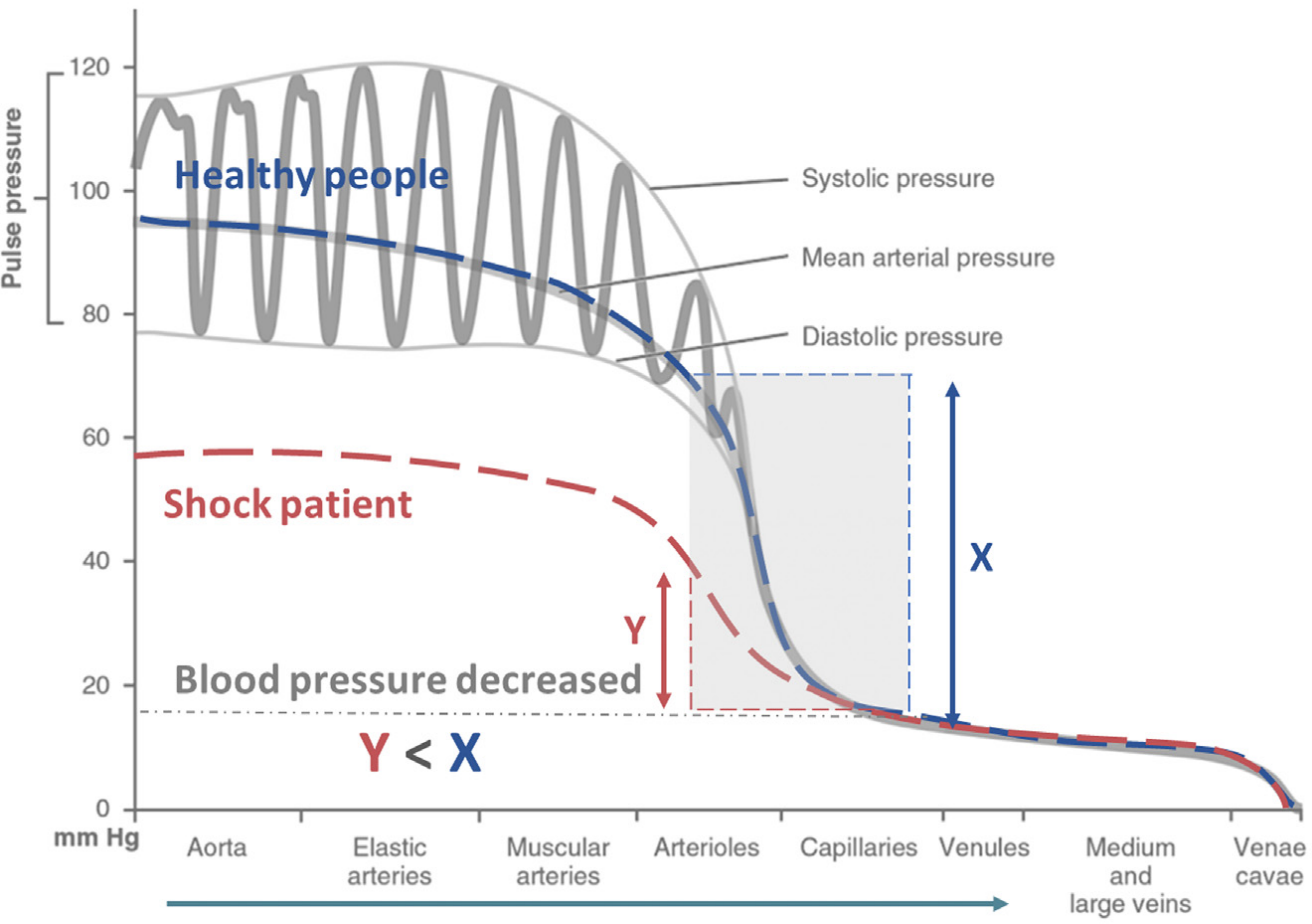
Graph of blood pressure changes in healthy subjects and shock patients This figure shows that while the blood pressures in the microvascular segments of the two groups of patients tended to be similar, the blood pressure in the proximal aorta of a patient experiencing shock was substantially lower than that of a healthy patient; as a result, it can be concluded that the change in flow velocity (X) of shock patients was lower than that of healthy subjects on the basis of the difference between the proximal blood pressure of shock patients and the coincident blood pressure (Y). According to this conclusion, a subject is highly likely to be in a state of shock when the proximal blood pressure is lower, and the difference between the proximal (in the knuckle) and distal (in the fingernail) measurements observed by LSCI is lower in a shock patient than the average value of healthy subjects.

According to hemodynamics, lower blood pressure results in a slower blood flow velocity. Blood pressure and flow velocity gradually decrease from the aorta to the microvasculature.^34^ As shown in Figure 5, the blood pressure of a shock patient is markedly lower than that of a healthy subject at the beginning of blood circulation (in the aorta), decreases toward the end of blood circulation and becomes more uniform in the microvasculature. Therefore, the difference in blood pressure between the initial blood pressure and the overlapping point in shock patients (Y) is smaller than the difference in healthy individuals (X). From this inference, it can be concluded that shock patients exhibit smaller changes in blood flow velocity. On the basis of this conclusion, using LSCI to observe lower differences in measurement values between the proximal (heart) and distal (limb peripheries) regions may indicate a lower blood pressure proximally or the potential risk of shock in the patient. In the case of shock, different tissues and organs may suffer from ischemia and hypoxia due to the body’s inability to maintain sufficient perfusion pressure, which leads to circulatory dysfunction. As the body tries to protect vital organs, peripheral blood perfusion is often reduced. Therefore, evaluating the condition of the peripheral blood circulation is important for the early diagnosis of shock. To validate this theoretical understanding and assess the effectiveness of LSCI in detecting peripheral blood circulation and identifying patients who are in shock, we conducted a comprehensive study in partnership with Changhua Christian Hospital. We collected physiological data, blood test results, and ROI diff values derived from LSCI measurements from 40 volunteers who worked at Changhua Christian Hospital between January 1 and December 30, 2022. Following the participants’ classification into shock-positive and shock-negative control groups, the data were subjected to numerous statistical analyses, such as ROC curve, boxplot, Spearman correlation, Mann‒Whitney U test, and chi-square tests. Using the Mann‒Whitney U test and the chi‒square test, *p* values were computed, indicating notable differences between the experimental and control groups in terms of physiological data as well as ROI diff values derived from the LSCI measurements. This finding suggests that the experimental and control groups differ in terms of ROI diff values and is consistent with the anticipated clinical presentations of shock patients.

The results from the boxplot analysis indicate that there is an overlap in the range of ROI diff data, which may lead to an increased risk of misdiagnosing shock. Additionally, Spearman’s correlation analysis revealed correlations between ROI diff values and physiological indicators such as HR, SBP, and MAP. These findings are consistent with the clinical features that are commonly observed in patients with shock. Using an ROC curve for the final examination, ROI diff was examined and found to have moderate accuracy in diagnosing shock. A specific threshold of ROI diff <6966.43 was selected to diagnose shock. After accounting for all the statistical findings, we can say that LSCI, in fact, offers a basis for diagnosing shock via the ROI diff values that are obtained. However, when LSCI is used to identify shock, the degree of data overlap could result in a higher misdiagnosis rate.

### More accurate criteria for shock diagnosis

Given the potential overlap in ROI diff data between shock and non-shock patients, more accurate diagnostic criteria for shock were needed. We statistically compared the ROI diff and ROI diff/ROI_2_ values to determine whether ROI diff/ROI_2_ was better at identifying shock patients to address the problem of overlapping ROI diff data between the experimental and control groups, which resulted in increased rates of misdiagnosis. First, the ROI diff and ROI diff/ROI_2_ values in the experimental and control groups were compared via the Mann‒Whitney U test; both produced *p* values less than 0.05, indicating significant differences. However, the boxplots revealed that the ROI diff/ROI_2_ data distribution did not overlap as much as the ROI diff data distribution did. This suggested that the rate of misdiagnosis due to overlapping data could be reduced if the ROI diff/ROI_2_ value was used as the basis for shock identification. The correlations between the ROI diff and ROI diff/ROI_2_ values and the physiological data were compared. Compared with the ROI diff value, the ROI diff/ROI_2_ value was not only correlated with SBP, MAP, and HR but also correlated with RR. Furthermore, the ROI diff/ROI_2_ value was more strongly correlated with SBP, HR, and RR. Finally, a comprehensive comparison of the ROI diff/ROI_2_ value with the ROI diff value was performed via the ROC curve and the confusion matrix to assess their accuracy. The ROI diff/ROI_2_ value had a greater AUC and a *p* value of less than 0.001, indicating a highly significant difference according to the validation results of the ROC curve. This outcome demonstrated that the ROI diff/ROI_2_ value provided better shock detection performance and justification. The associated criterion, which was determined from the ROC curve and used as a reference point for the use of the ROI diff and ROI diff/ROI_2_ values in diagnosing shock, was then used to create a confusion matrix. It is evident from the results of the confusion matrix analysis that using the ROI diff/ROI_2_ value rather than the ROI diff value for shock diagnosis yields better results in terms of accuracy, recall, and F1 score. This means that the ROI diff/ROI_2_ value can identify shock more accurately and demonstrates better performance in shock diagnosis. On the basis of these statistical results, when the ROI diff/ROI_2_ value is used as a reference, LSCI can be used to determine whether a patient is in shock by measuring the blood volume in the fingertip.

### Comparison of the developed LSCI technology and conventional methods for shock diagnosis

To comprehensively assess the performance of LSCI for detecting shock, we first evaluated commonly used shock detection parameters, including MAP, SBP, HR, and SpO_2_ (see Figure S1). The comprehensive evaluation of these common shock detection standards supported their integration into clinical protocols for early and accurate shock diagnosis. While the AUC value for ROI diff/ROI_2_ was slightly lower than that for some traditional methods, LSCI technology offers significant advantages in rapid and noninvasive blood flow change measurements.

By comparing the ROI diff/ROI_2_ values obtained by observing patients with LSCI and the results of conventional SI, we found that LSCI could comprehensively and rapidly detect microcirculatory changes with high sensitivity, as shown by the correlation and accuracy of the physiological data; these results highlight the advantages of LSCI technology. First, the feasibility and benefits of LSCI in different groups were verified via systematic data analysis. Compared with conventional SI, the LSCI technique provides more evaluative information, including a quantitative assessment of microcirculatory abnormalities, blood flow velocity, and vascular density, which are important indicators of pre-shock onset. Furthermore, regarding the correlation with physiological data, we observed that the ROI diff and ROI diff/ROI_2_ values obtained with LSCI were correlated to a certain degree with the participants' physiological parameters, such as SBP, MAP, and HR. This finding indicates that LSCI technology has the ability to reflect the physiological status of patients.

Furthermore, in the ROC curve analysis, our study revealed that the shock indicators that were obtained by LSCI (ROI diff and ROI diff/ROI_2_) exhibited high accuracy, with values of 72.5% and 82.5%, respectively; these results demonstrated the substantial effectiveness of LSCI in distinguishing between shock and non-shock patients. In terms of measurement speed, convenience, and hygiene, LSCI technology has important advantages. Previously, obtaining SI values required simultaneous data about blood pressure and heart rate. Traditional blood pressure monitors and heart rate monitors typically require more than a minute to collect measurements, and both methods require contact with the patient. Traditional blood pressure monitors also have the disadvantage of being unable to provide real-time measurements. However, as shown in Figure 4, LSCI, whether the ROI diff or ROI diff/ROI_2_ value is used as an indicator, can be used to diagnose whether a patient is experiencing shock with a certain level of accuracy in a short time, i.e., as little as 30 s. LSCI can also continuously monitor a patient’s current blood circulation status in real time, demonstrating the stability and rapid imaging capabilities of LSCI technology. In addition, measurements can be conducted without direct contact with the patient, further reducing hygiene concerns.

The findings of this study confirm that LSCI technology offers rapid, noninvasive, contactless, and continuous monitoring capabilities. The speed at which LSCI provides critical information can allow healthcare professionals to obtain vital microcirculation indicators, significantly improving the real-time monitoring of a patient’s condition. Its noninvasive and contactless nature offers a comfortable testing experience for patients while reducing the risk of cross-infections. Compared with traditional methods, LSCI delivers more comprehensive assessment criteria and demonstrates strong correlations with certain physiological parameters and accuracy. Additionally, the ability of LSCI to complete blood flow measurements within 30 s is crucial for managing acute shock cases, providing real-time insights into microcirculatory changes, especially in situations where patient contact is not possible or traditional methods cannot be applied, such as open trauma or extensive burns. Traditional shock assessment methods, such as the measurement of MAP, SBP, HR, and SpO_2,_ remain widely used in clinical settings because of their reliability in providing physiological information. However, these methods often require direct patient contact and, in some cases, are invasive (e.g., arterial catheter pressure measurement), which can increase the risk of infection, particularly in patients with severe trauma or burns. Furthermore, traditional methods tend to be slower, potentially delaying diagnosis and treatment. In contrast, the rapid and noncontact nature of LSCI makes it an effective supplementary tool, allowing for quick decision-making in clinical scenarios requiring immediate action. Nevertheless, LSCI also has limitations. While it can provide accurate blood flow information rapidly, its ability to assess the hemodynamics of deeper vessels is less advanced than that of imaging technologies such as CT and MRI, which offer more detailed views of tissues and organs. Additionally, LSCI data may be influenced by environmental lighting conditions, although we mitigated this influence in this study by normalizing the ROI values. Compared with traditional shock indicators such as the SI and CRT, the indicators obtained with LSCI have significant advantages. Although the SI is widely used in clinical practice, LSCI provides more comprehensive assessment criteria, particularly for microcirculation research. Similarly, both the CRT and LSCI can be used to detect shock rapidly, but numerous studies have indicated that the CRT can be affected by subjective influence, is difficult to quantify, and currently cannot be continuously monitored in real time,^35^^,^^36^ highlighting the clear advantages of LSCI.

This capability is crucial in clinical situations requiring quick decision-making, such as during surgical procedures or in emergency rooms. The rapid detection capability of LSCI makes it a valuable supplementary tool for diagnosing shock, particularly in scenarios where immediate blood flow information is needed. LSCI not only provides a foundation for future research on microcirculation but also offers clinicians a powerful tool for more accurately assessing the blood circulation and predicting shock. Compared with traditional methods, the speed and noncontact nature of LSCI make it a promising supplementary tool, improving the efficiency of early shock diagnosis and supporting rapid clinical decision-making.

### Advantages and limitations of LSCI in diagnosing shock

**Figure 6:**
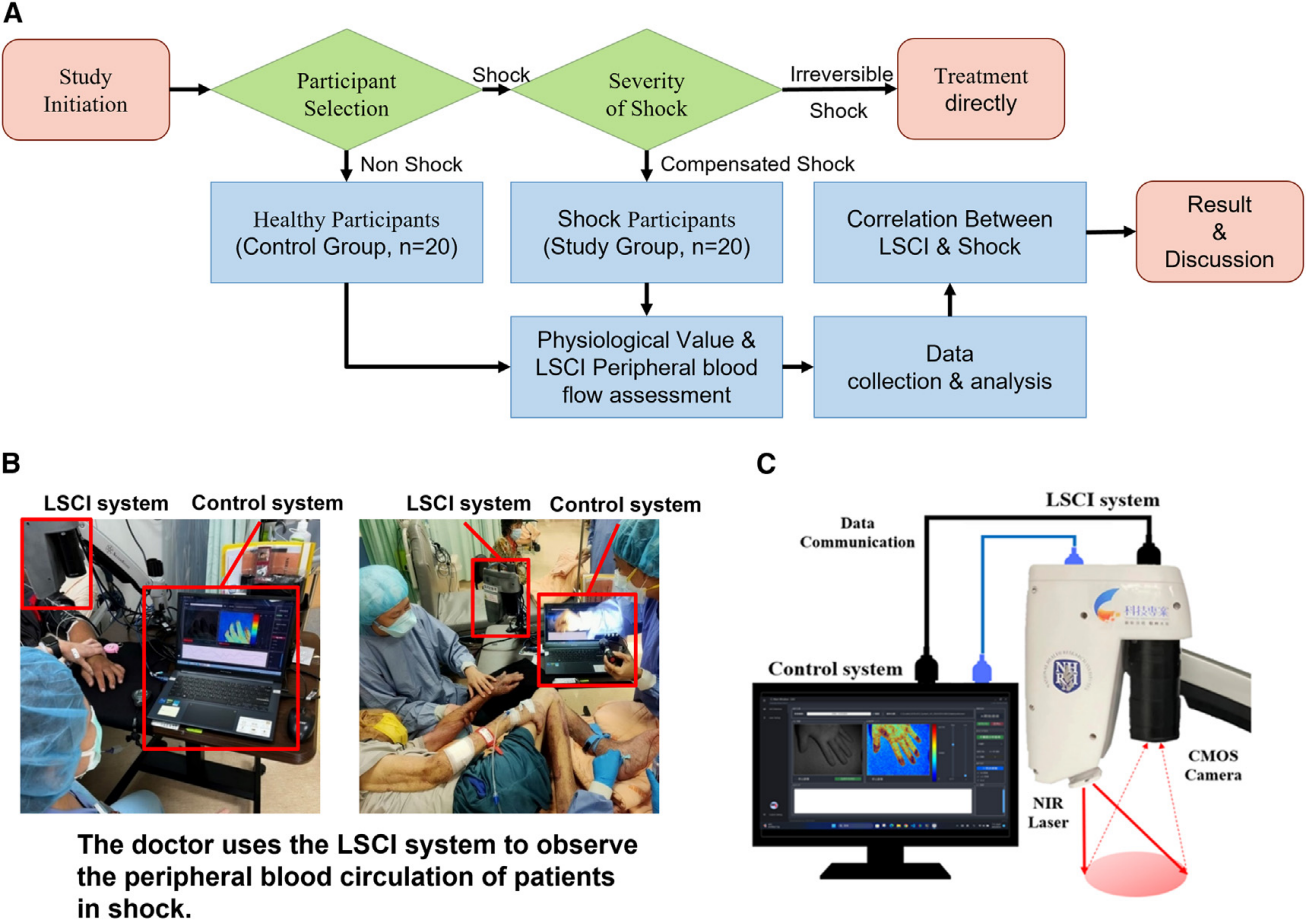
Shock detection process and system architecture of LSCI technology (A) The experimental flow chart; the participants were classified by medical personnel, with non-shock participants assigned to the control group (*n* = 20). If a participant was identified as a shock patient, the severity of shock was immediately assessed. To ensure the safety of the participants, the experiment excluded individuals with severe shock conditions such as trauma, peripheral vascular disease, or chronic wounds. When medical personnel determined that a patient had experienced early-stage shock, consent was obtained from the patient’s family, and the patient was assigned to the experimental group (*n* = 20). Subsequently, physiological parameter measurements and blood tests of the LSCI were conducted; after data collection, statistical analysis was performed to assess the usability of the data. The study concluded with a detailed explanation of the correlation between the LSCI results and shock parameters as well as the effectiveness of LSCI in identifying people in shock. (B) The illustration shows the physician illuminating a patient’s hand in a clinical environment utilizing LSCI to collect blood flow data in the region of interest (ROI); the benefits of the LSCI system, including its noninvasiveness, real-time feedback, and ease of use, are demonstrated in this study. (C) The LSCI system architecture is shown. An 805 nm operating near-infrared semiconductor laser diode serves as the laser system’s radiation source; the output energy is regulated by the laser diode driver; an optical diffuser is positioned at the laser output and scatters light at a 50° angle to ensure uniform dispersion of the light energy and increase the illuminated area; an array scanning camera, a monochrome camera with a CMOS sensor that can capture images with resolutions of up to 1280 × 1024 pixels, is used for image acquisition; and a bandpass filter is positioned in front of the lens to exclude light sources with wavelengths shorter than 805 nm and guarantee that only images created by laser speckle are recorded.

**Figure 7:**
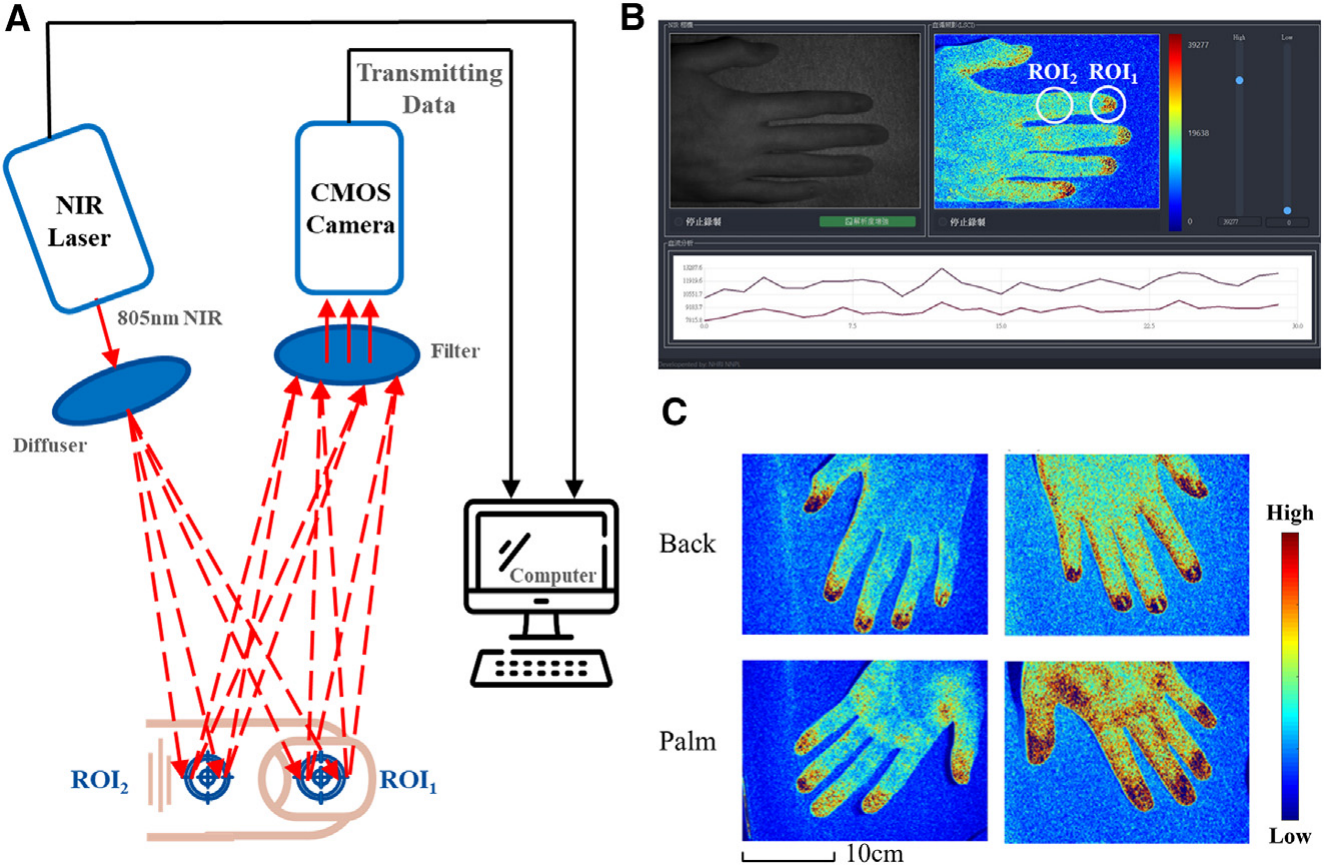
Clinical operation and peripheral blood flow detection with LSCI (A) A schematic diagram of the LSCI operating system is shown. At the time of detection, the index finger circles were ROI_1_ (nail) and ROI_2_ (skin). The NIR laser source and CMOS sensor were controlled and switched on and off by a computerized device. The laser light source emitted near-infrared light with a wavelength of 805 nm, and a diffusion device was used to reduce the intensity of the light source and increase the area covered by the light source. A scattered light source with a wavelength of 805 nm was collected by a filtering device, and the data were transmitted to a computer device through a transmission line to record the ROI values, with a focus on the fingernail and the skin of the index finger. The difference in the ROI value was obtained by subtracting ROI_1_ and ROI_2_, and the difference in the ROI was used to determine blood flow in the test area. (B) The actual LSCI operation screen is shown. After the LSCI was activated, the LSCI control system was turned on, and a circle with a radius of 20 mm was used as the focus detection area. The ROI_1_ and ROI_2_ values of the index finger were circled, and then the changes in the ROI values could be observed by the line graph below. (C) LSCI was performed on the palms and backs of the hands. As shown in the figures, blood flow was most abundant at the fingertips, indicating prominent blood circulation in this region; this phenomenon is attributed to the presence of a substantial capillary bed formed by numerous microvessels at the fingertips, leading to increased blood flow detected by LSCI. Consequently, we selected the regions of interest (ROIs) for analysis as the nail bed of the index finger (ROI_1_) and the skin at the fingertip (ROI_2_) during the experimental procedure.

LSCI demonstrates several key advantages in diagnosing shock, particularly in its ability to provide rapid, noncontact assessments of peripheral blood flow. Our established shock detection process and LSCI system architecture (Figure 6) enable efficient clinical implementation, while the standardized operation protocol for peripheral blood flow detection (Figure 7) ensures consistent and reliable measurements. In the clinical diagnosis of shock, medical personnel first evaluate a patient’s SBP, MAP, and lactate concentration and then evaluate whether any of these three indicators exceeds the clinical criteria for shock diagnosis to determine whether the patient is experiencing shock. However, three of the twenty patients in this clinical trial who were experiencing shock did not meet the requirements for shock diagnosis, including lactate concentration, MAP, or SBP, as shown in Table 1. Subsequently, LSCI was used to measure the ROI diff and ROI diff/ROI_2_ values of each of the three individuals, and the results fell within the shock threshold range. Compared with the use of blood pressure and lactate concentration to diagnose shock, although LSCI cannot be used to determine whether a patient is experiencing shock, it can save time by increasing measurement accuracy and decreasing the need for multiple instruments during the initial detection process.

Although SBP and MAP demonstrated higher accuracy than LSCI for diagnosing shock, both parameters failed to correctly identify certain shock patients (As shown in Figure S2). Our study found that the MAP model missed 12 patients, while LSCI successfully identified 7 of these as potential shock cases. Similarly, SBP missed 9 patients, with LSCI successfully identifying 7 of these as potential shock cases. These results highlight LSCI’s advantage as an auxiliary tool that is capable of identifying potential shock patients missed by conventional methods. This finding suggests that LSCI may provide additional diagnostic information in situations where traditional methods may lack sensitivity. However, it should be noted that the criteria for shock diagnosis may vary due to the use of different LSCI instruments and environmental conditions. Additionally, the advantage of LSCI lies in its ability to be used effectively in a variety of clinical conditions. This clinical trial demonstrated the effectiveness of LSCI in identifying shock. Compared with traditional shock indicators and methods, LSCI has the advantages of rapid and real-time monitoring, and more importantly, it can be used to diagnose patients without touching them, extending its applicability beyond general shock patients. Our comprehensive study encompassed various aspects of LSCI’s performance in shock detection. We evaluated LSCI across different types of shock and conducted comparative analyses with traditional shock detection methods. The results consistently showed that both ROI diff and ROI diff/ROI_2_ values were lower in the shock group than in the control group across all shock types. This finding underscores the robustness of LSCI in detecting shock across various clinical presentations. Furthermore, we performed extensive comparisons between LSCI and traditional methods such as MAP, SBP, HR, and SpO_2_ using ROC curve analyses and confusion matrices. These comparisons provided valuable insights into the relative strengths of LSCI and conventional shock detection techniques. Notably, our investigation also extended to analyzing HRV parameters, revealing significant differences between shock patients and the control group. This observation opens up new avenues for understanding the physiological responses in shock and potentially enhances our diagnostic capabilities. Even for patients with extensive trauma where contact-based detection is not possible or for patients with multiple types of trauma, LSCI enables frontline medical personnel to examine these patients more easily, as it reduces testing time by more than 50% compared with traditional methods and eliminates the need for complex professional operations. The second phase of our study further confirmed the advantages of LSCI for detecting various types of shock (see Table S1). Importantly, this phase demonstrated the ability of LSCI to quickly identify potential shock patients regardless of the shock type. This finding has significant clinical implications, suggesting that LSCI could be a versatile tool for detecting various types of shock. The multi-functionality of LSCI enables healthcare providers to make timely and beneficial medical decisions, potentially improving patient outcomes. This is particularly crucial in emergency and intensive care settings where rapid and accurate diagnosis is vital.

A growing body of research has shown that LSCI holds significant potential in noninvasive medical diagnostics, particularly in peripheral hemodynamic monitoring. For instance, Sdobnov et al.^21^ highlighted the role of LSCI in visualizing cerebral hemodynamics, especially during and after cardiac arrest. Zherebtsov et al.^22^ validated the clinical utility of LSCI in detecting microvascular changes associated with aging and diabetes. Additionally, Zharkikh et al.^23^ reviewed the application of LSCI in detecting diabetic complications, whereas Kalchenko et al.^24^ explored its strengths and limitations in brain imaging. These studies underscore the value of LSCI as a real-time clinical feedback tool and its applications across various medical fields. Future research will expand its use in shock diagnostics and other critical conditions, promoting broader clinical integration.

### Integration of HRV analysis with LSCI

To further enhance the diagnostic capabilities of LSCI and gain deeper insights into the physiological responses during shock, we incorporated HRV analysis in the second phase of our study (As shown in Table S2). This integration provided new perspectives on the complex cardiovascular dynamics associated with shock states.

The second phase of our study incorporated HRV analysis, providing new insights into physiological responses during shock. Interestingly, the HRV of the shock group was greater than that of the control group, potentially indicating a more adaptive and resilient autonomic nervous system in shock patients. The greater variability and complexity in heart rate dynamics observed in the shock group could reflect the body’s attempt to maintain homeostasis under stress. Significant differences in frequency domain measures suggest variations in autonomic regulation between the groups, which may be a compensatory mechanism in shock states. These HRV findings have important implications for understanding the physiological differences between shock and non-shock states. They highlight the potential of HRV analysis as a complementary tool to LSCI in shock assessment, thus offering a more comprehensive view of cardiovascular dynamics during shock. Future studies could explore how these HRV changes correlate with LSCI measurements and clinical outcomes, potentially leading to more nuanced shock detection and management strategies. The integration of HRV analysis with LSCI represents a significant step toward a multi-modal approach to shock assessment, potentially improving the overall accuracy and timeliness of shock diagnosis, especially in complex or ambiguous clinical scenarios.

### Conclusion

This research proposes a new and practical method for the use of LSCI technology, which can monitor the peripheral circulatory state of patients and detect early warning signals of shock. LSCI technology allows researchers to obtain accurate and real-time data about a patient’s microcirculation without invasive procedures. This technique not only provides numerical assessments of vessel density and blood flow velocity but also identifies anomalies in the microcirculation that are considered crucial markers of the pre-shock stage. This study examined the feasibility and benefits of using LSCI technology in clinical diagnosis by analyzing its performance in different patient groups. The results of the Mann‒Whitney U test revealed that there was a significant difference in the distributions of “ROI diff” and “ROI diff/ROI_2_” between the experimental and control groups (both with *p* < 0.05). Spearman’s correlation analysis confirmed the correlations of the ROI diff value with SBP, MAP, and HR, which was consistent with the clinical performance characteristics of shock patients. Furthermore, ROC curve analysis revealed that the ROI diff and ROI diff/ROI_2_ values can be used to distinguish shock patients from non-shock patients (with accuracies of 72.5% and 82.5%, respectively). The use of LSCI technology can provide vital support for clinicians in making decisions, preventing shock, and detecting early warning signs. LSCI also provides a new perspective and opportunity for future studies on peripheral blood circulation and associated methodologies. This study highlights the potential applications of LSCI technology in clinical care and biomedical engineering. After conducting a thorough investigation and evaluation of different image processing and analysis techniques, we gained valuable insights into how this technology can improve patient care and clinical diagnosis. The outcome of our work provides a strong basis for further research in this field and encourages innovative and practical applications in microcirculation research.

### Limitations of the study

This study utilized LSCI to assess peripheral blood flow in shock patients. Although LSCI demonstrated excellent performance in detecting peripheral hemodynamic changes and providing rapid, noncontact assessments, there were some limitations. First, due to the small sample size and lack of consideration for gender and ethnic differences, the results may not fully represent the broader population’s responses. Although the supplementary research covered different types of shock, these data are preliminary and further validation is needed in larger and more diverse cohorts. Additionally, the impact of using different LSCI instruments and environmental conditions on measurement outcomes should be noted, suggesting the need for standardized protocols to ensure consistency in clinical applications. Despite these limitations, this study highlights the potential value of LSCI for diagnosing shock, especially in complex clinical scenarios.

## Resource availability

### Lead contact

Further information and requests for resources and reagents should be directed to and will be fulfilled by the lead contact, Lun-De Liao (ldliao@nhri.edu.tw).

### Materials availability

This study did not generate new unique reagents.

### Data and code availability

• Original LSC images reported in this paper will be shared by the lead contact upon request.
• All original code will be shared by the lead contact upon reasonable request.
• Any additional information required to reanalyze the data reported in this paper is available from the lead contact Lun-De Liao (ldliao@nhri.edu.tw) upon request.
• Two datasets have been deposited: Figshare: Dataset (IRB number: 211116), Figshare: Dataset (IRB number: 230705).

## Acknowledgments

This research was supported in part by the 10.13039/100020595National Science and Technology Council of Taiwan under grant numbers 110-2221-E-400-003-MY3, 111-3114-8-400-001, 111-2314-B-075-006, 111-2221-E-035-015, 113-2221-E-400-003, 111-2221-E-005-018, 112-2221-E-005-042 and 113-2221-E-005-011 and by the 10.13039/501100004737National Health Research Institutes of Taiwan under grant numbers NHRI-EX108-10829EI, NHRI-EX111-11111EI, and NHRI-EX111-11129EI. Meng-Che Hsieh carried out this research with funding support in part from the Doctoral Program in Tissue Engineering and Regenerative Medicine of National Chung Hsing University and 10.13039/501100004737National Health Research Institutes.

## Author contributions

M.-C.H., L.-D.L., Y.-R.L., and P.-Y.H. jointly conceived the project and designed the experiments. M.-C.H. and S.-Y.L. prepared the experimental equipment, while Y.-R.L. and P.-Y.H. conducted the experiments. M.-C.H., Y.-R.L., P.-Y.H., and S.-Y.L. analyzed the data. M.-C.H., S.-Y.L., and L.-D.L. wrote the manuscript. J.-J.H. and C.T.S.C. reviewed and revised the manuscript.

## Declaration of interests

The authors declare no conflicts of interest.

## STAR★Methods

### Key resources table

**Table:** 

REAGENT or RESOURCE	SOURCE	IDENTIFIER
**Software and algorithms**		

MedCalc	MedCalc Software Ltd, Ostend, Belgium	https://www.medcalc.org/
Excel LTSC Professional Plus 2021	Microsoft, Seattle, Washington, USA	https://www.microsoft.com/zh-tw/microsoft-365
LSCI program system	Hsieh et al.^37^	https://doi.org/10.1063/5.0172443

**Deposited data**		

Figshare: Dataset (IRB number: 211116)	Lun-De Liao	https://doi.org/10.6084/m9.figshare.27012088.v2
Figshare: Dataset (IRB number: 230705)	Lun-De Liao	https://doi.org/10.6084/m9.figshare.27018562.v1

### Experimental model and study participant details

#### Setting and study population

This study was approved by the Institutional Review Board (IRB) of Changhua Christian Hospital under two separate protocols (IRB numbers: 211116 and 230705). The research was conducted in two phases, with experimental data collected at Changhua Christian Hospital in Taiwan. It should be noted that the main content of this paper focuses on the first phase of the trial, while the second phase yielded supplementary data.

The first phase (IRB number: 211116) is the core of this research and aimed to comprehensively evaluate the effectiveness of LSCI in identifying shock patients. The primary purpose of this phase was to establish standards for shock identification using LSCI and to conduct an in-depth comparison of physiological data between the experimental and control groups. The research focused on exploring the application of ROI diff and ROI diff/ROI_2_ values in shock detection, while also analyzing the performance of LSCI in identifying shock across different time windows. Furthermore, this phase assessed the potential clinical application of LSCI as a diagnostic tool for shock. This phase recruited 40 Chinese participants residing in Taiwan. Among them, 20 participants exhibiting shock symptoms formed the experimental group, while the other 20 healthy individuals served as the control group. The shock group included 14 males and 6 females, while the control group comprised 8 males and 12 females. All shock participants, some of whom were elderly, were admitted to the hospital under emergency conditions. The majority of these patients had at least one chronic underlying condition, including hypertension (H.T.N.), diabetes mellitus (DM), or cardiovascular disease (CVD). More detailed information can be found in Table 1.

The second phase (IRB number: 230705) served as a supplementary study and to obtain further knowledge about the application of LSCI in shock diagnosis. This phase primarily focused on studying the performance of LSCI across different types of shock while introducing electrocardiogram (ECG) measurements as an auxiliary standard for shock assessment. The study analyzed heart rate variability (HRV) performance in shock patients and explored the correlation between LSCI and HRV parameters. Through these additional measurements and analyses, the second phase trial further validated the reliability of LSCI in diagnosing shock. This phase also included 40 participants, with 20 shock patients in the study group and 20 healthy individuals in the control group. In this phase, the shock and control groups consisted of 9 males and 11 females. Similar to the first phase, shock patients in the second phase also had various chronic underlying diseases, the specific composition of which can be found in Table S1.

In both phases, healthcare professionals screened shock patients who had not received inotropic or vasoconstrictor agents based on early shock symptoms (systolic blood pressure <95 mmHg, mean arterial pressure <65 mmHg, and lactate concentration >2 mmol/L) and clinical manifestations of shock. For safety considerations, both studies excluded patients with trauma, peripheral vascular diseases, chronic wounds, and other severe cases of shock.

The research procedure was generally similar in both phases. When clinical physicians diagnosed patients with shock and their conditions had stabilized, the research team obtained consent from the patients' families. Subsequently, researchers immediately used LSCI to record patients' ROI data. In the second phase, LSCI measurements were conducted simultaneously with ECG measurements to collect ROI data and electrocardiogram data concurrently. This synchronized measurement method allowed us to calculate HRV-related parameters and examine their correlation with LSCI data, thereby comprehensively assessing shock patients' hemodynamics and autonomic nervous system function.

All participants and their families were informed about the experimental plan and signed informed consent forms. Although sex was recorded for all participants, neither study specifically analyzed the influence of sex on the results, which remains a limitation of the research. The combined objective of these two phases was to comprehensively evaluate the effectiveness and reliability of LSCI in identifying and diagnosing different types of shock patients, and to explore the correlation between LSCI and other physiological parameters, thus providing a scientific basis for clinical applications.

### Method details

#### Experimental methods

This study recruited a total of 80 participants across two phases, with 40 participants in each phase. The grouping and experimental procedures were performed according to the flow chart in Figure 6A. In both phases, LSCI was employed to monitor each participant continuously for 60 seconds, with an average sampling rate of 2.7 Hz. Figure 6B illustrates the collaborative use of LSCI by healthcare professionals to detect shock in participants. All participants underwent LSCI examination without direct contact with the device. To determine the regions of interest (ROIs) during LSCI testing, we conducted preliminary experiments to identify the optimal observation areas. As shown in Figure 7C, blood flow patterns on both the back of the hand and the palm exhibited prominent blood flow at the fingertips. Blood circulation is most pronounced in the fingers; capillary beds in the finger tips are characterized by shallow and dense microvessels, making them highly responsive to changes in blood circulation.^38^^,^^39^ This observation aligns with findings by Ruan et al.*,*^40^ who reported significant changes in the perfusion index (PI) in the fingertips of patients with septic shock, highlighting the sensitivity of fingertips compared with that of the less responsive finger joints. Based on these observations, during the enrollment process, ROIs in the fingernail area (ROI_1_) and the skin on the fingertip (ROI_2_) of the index finger or middle finger were selected, as shown in Figures 7A and 7B. When healthcare professionals placed a participant's hand in the sensing area and activated the LSCI system, they selected these key observation points. It was essential to verify that the chosen detection area did not extend beyond the fingertip to avoid inaccuracies in the test results. After a continuous 60-second recording, the values obtained were stored in a folder. The subsequent calculation involved subtracting ROI_2_ from ROI_1_ to derive the ROI difference (ROI diff), which was used to assess blood flow. Additionally, we normalized the ROI diff by dividing it by ROI_2_ (ROI diff/ROI_2_), using ROI_2_ as a reference point, to mitigate potential variations in light interference during clinical measurements due to different environmental conditions.

In the first phase, various physiological parameters (blood pressure, BT, HR, RR, SpO_2_, CBC) were measured after LSCI. In the second phase, in addition to LSCI measurements, we simultaneously conducted ECG measurements. The ECG device was attached to a different finger not observed by LSCI, allowing us to gather complementary data. This ECG measurement was conducted for approximately 90 seconds, providing data for heart rate variability (HRV) analysis.

After completing the data collection for all participants in both phases, analyses were performed to validate whether the ROI diff values in the experimental and control groups were correlated with changes in blood flow during shock. Additionally, correlation analyses were performed to examine the associations between the ROI diff values, physiological parameters, and HRV parameters (in the second phase). This comprehensive approach allowed us to not only assess the effectiveness of LSCI in identifying shock but also to explore its relationship with other physiological indicators, particularly HRV, thus providing a more holistic view of shock diagnosis and monitoring.

#### LSCI principles

Laser speckle is a random intensity distribution formed due to the scattering of light in a medium. When a camera captures these speckle patterns, the resulting image appears blurred if the exposure time exceeds the speckle decorrelation time. This blur is quantified by the speckle contrast K. In accordance with Goodman,^41^ the speckle contrast K is defined as the ratio of the standard deviation σ of the speckle intensity to its mean intensity Equation 1:

(Equation 1) $$K=\frac{\sigma }{\left\langle I\right\rangle }=\frac{\sqrt{\left\langle I^{2}\right\rangle -{\left\langle I\right\rangle }^{2}}}{\left\langle I\right\rangle }$$

In a fully developed speckle pattern, K=1; in dynamic speckles, K decreases, indicating increased blurring. Theoretically, K ranges from 0 to 1, where 0 indicates rapid particle movement, causing complete speckle blurring.

Fercher and Briers^37^ proposed a formula for calculating speckle contrast in a single exposure based on assumptions about the particle velocity distribution:

(Equation 2) $$K=\sqrt{\frac{\tau_{c}}{2T}\left[1-\exp\left(-\frac{2T}{\tau_{c}}\right)\right]}$$

In Equation 2, T is the camera exposure time, and $\tau_{c}$ is the speckle correlation time, reflecting the particle speed.

Furthermore, Bandyopadhyay et al.^42^ introduced a corrected formula that accounts for the speckle averaging effect:

(Equation 3) $$K=\sqrt{\beta \left\{\frac{\tau_{c}}{T}+\frac{\tau_{c}^{2}}{2T^{2}}\left[\exp\left(\frac{-2T}{\tau_{c}}\right)-1\right]\right\}}$$

In Equation 3, β is a correction factor included to accommodate differences in experimental conditions

The velocity $v_{c}$ of the scattering particles can be estimated on the basis of $\tau_{c}$ and is related to the wavelength λ:

(Equation 4) $$v_{c}=\frac{\lambda }{2\pi \tau_{c}}$$

The speckle contrast K and the particle speed $v_{c}$ demonstrate an inverse square relationship, that is:

(Equation 5) $$\frac{1}{K^{2}}\propto v$$

These theoretical models suggest that LSCI can be used an effective method for measuring relative blood flow velocities. However, the simplifications of the equations, which are based on numerous assumptions, restrict the use of LSCI primarily to relative assessments of blood flow rather than the determination of absolute measurements.

#### LSCI system and operation

To obtain real-time speckle images to analyze the relationship between changes in blood flow velocity and LSCI results, we utilized the principles of LSCI and spatial speckle contrast analysis. This research is based on the foundational theories proposed by Goodman, Fercher, Briers, Bandyopadhyay, and others.^37^^,^^41^^,^^42^^,^^43^^,^^44^^,^^45^ Measurements were conducted via the LSCI instrument that was developed in this study, which is illustrated in Figure 6C. The instrument comprises a semiconductor laser light source that emits near-infrared (NIR) light at a wavelength of 805 nm and a power of 500 mW (ML620G40, Thorlabs, USA). To enable the penetration of light into human skin, an integrated laser diode driver (SF8075-NM, Maiman Electronics, Russia) is used in conjunction with control software to adjust the output energy. A circular diffuser (ED1-C50, Thorlabs, USA) attached to the laser output scatters light at a 50° angle to increase the illuminated area on the target and ensure equal distribution. The area scan camera (acA1300-200μm, Basler, Germany) used in the image acquisition system has a complementary metal‒oxide‒semiconductor (CMOS) sensor and can take pictures with a resolution of 1280 × 1024 pixels. To achieve optical magnification ranging from 0.084× to 0.84×, we utilized a macrozoom lens (MLH-10×, Computar, Japan) that offers a focal length of 0.15 to 0.45 m and an aperture ranging from F5.6 to F32C. This lens provides flexible adjustment options for magnification, zoom, and focus, allowing it to seamlessly adapt to varying focal lengths across different operational distances and imaging scenarios. During measurement, the distance between the lens and the target was maintained at 30 cm, and the energy was uniformly dispersed through a diffuser within the imaging area of 20×15 cm. A total of 96.7% of the field of view (FoV) falls within the full width at half maximum (FWHM), with a power range of 238.3 to 525.1 μW reaching the target. A bandpass filter is placed in front of the lens to reduce interference and mistakes caused by outside light. This ensures that the images that are captured are solely the result of the NIR laser (805 nm) light reaching the CMOS sensor. The area scan camera, with an exposure time of 5 ms and a frame rate of 15 fps for each flow speed, transmits the received data to a computer for computation to visualize the blood circulation in the illuminated area. The LSCI program system^46^ is designed to track the target patient, can perform real-time perfusion calculations over a wide region, and allows users to adjust the laser light source and image sensor. Through this system, users can observe real-time images, control the laser output, and select ROIs to record and observe the blood flow data. The recorded data, including NIR images, LSCI images, and perfusion data, are stored as .csv files.

### Quantification and statistical analysis

The physiological data and various participant test results are presented as the mean ± standard deviation, median, and quantity (%). Differences in the means and standard deviations were used to assess the normality of the data distribution. For the analysis of the ROI diff, systolic blood pressure (SBP), diastolic blood pressure (DBP), mean arterial pressure (MAP), body temperature (BT), heart rate (HR), respiratory rate (RR), peripheral oxygen saturation (SpO_2_), height, and weight, the Mann‒Whitney U test and Z test were used to determine whether there were differences in physiological values between the experimental and control groups, with a predefined significance level of α equal to 0.05. Cohen’s *d* was calculated to assess the effect size of these differences, providing an indication of the practical significance of the results (as shown in Tables 1 and S1). Additionally, box-and-whisker plots were used to visually represent the physiological data for both the study group and the control group, along with the corresponding *p* values. Correlations that were significant at the 0.01 level are marked with two asterisks (∗∗), and those significant at the 0.05 level are marked with a single asterisk (∗) (see Figure 1).

Spearman’s rank correlation analysis was performed to analyze complete blood count (CBC) and biochemical results, including white blood cell count (WBC), hemoglobin (Hb), hematocrit (Hct), platelet count (PLT), glucose, sodium ion, phosphate ion, lactate (Lac), potassium ion, alanine transaminase (GPT), PCO_2_, and PO_2_, within the experimental group. With this analysis, we sought to determine whether there was a correlation between ROI diff and blood tests. Correlations that were significant at the 0.01 level are marked with two asterisks (∗∗), and those significant at the 0.05 level are marked with a single asterisk (∗) (as shown in Figure 2).

ROC curves were plotted for both the ROI diff and ROI diff/ROI_2_ values (as shown in Figure 3), the analysis of ROI diff and ROI diff/ROI_2_ over different periods (as shown in Figure 4), and the analysis of MAP and SBP (as shown in Figure S1). The analysis included parameters such as the area under the curve (AUC), 95% confidence interval (CI), *p* value, and associated criterion. These metrics were used to evaluate the accuracy, reliability and threshold values of LSCI in the identification of shock patients. The associated criteria obtained from the ROC curves were the basis for determining shock. The confusion matrix, accuracy, recall, and F1 score for the ROI diff and ROI diff/ROI_2_ values were calculated, providing a comprehensive assessment of the performance of LSCI in the identification of shock. A comparison of the accuracy of the ROI diff and ROI diff/ROI_2_ values in identifying shock patients was conducted.

Statistical analysis was performed via MedCalc Statistical Software (MedCalc 2023, Ostend, Belgium), and MS Excel (Excel LTSC Professional Plus 2021; Microsoft, Seattle, Washington, USA) was used for the statistical calculations.
