# Supplementary material for: Improving the early diagnosis and clinical outcomes of shock patients via laser speckle contrast imaging assessment of peripheral hemodynamics
Source: iScience. 2024 Nov 4;27(12):111307. doi: 10.1016/j.isci.2024.111307 (PMC11681897; doi:10.1016/j.isci.2024.111307)
Supplement: Document S1. Figures S1 and S2 and Tables S1 and S2 [file mmc1.pdf]

iScience, Volume *27*

Supplemental information

Improving the early diagnosis and clinical outcomes of shock patients via laser speckle contrast imaging assessment of peripheral hemodynamics

Meng-Che Hsieh, Jin-Jia Hu, Yan-Ren Lin, Shih-Yu Li, Pei-You Hsieh, Congo Tak Shing Ching, and Lun-De Liao

Supplementary figures

(A)

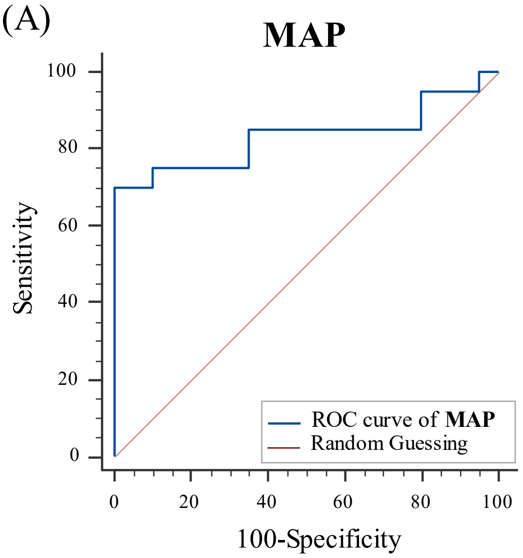

AUC	0.833
p-value	< 0.0001
95% CI	0.681~0.932

Accuracy	85.00%
Recall	70.00%
F1 score	82.35%

MAP(n=40)	Positive(P)	Negative(not P)
Shock	true positive=14	false negative=6
Non-Shock	false positive=0	true negative=20

(B)

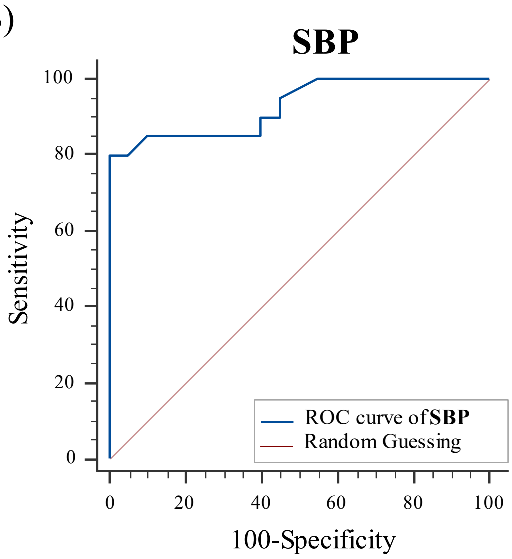

AUC	0.929
p-value	< 0.01
95% CI	0.801~0.986

Accuracy	90.00%
Recall	80.00%
F1 score	88.89%

SBP(n=40)	Positive(P)	Negative(not P)
Shock	true positive=16	false negative=4
Non-Shock	false positive=0	true negative=20

(C)

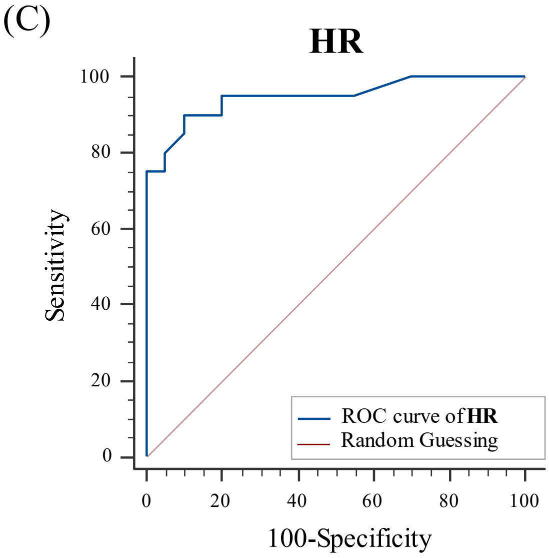

AUC	0.947
p-value	< 0.001
95% CI	0.827~0.993

Accuracy	90.00%
Recall	90.00%
F1 score	90.00%

HR(n=40)	Positive(P)	Negative(not P)
Shock	true positive=18	false negative=2
Non-Shock	false positive=2	true negative=18

(D)

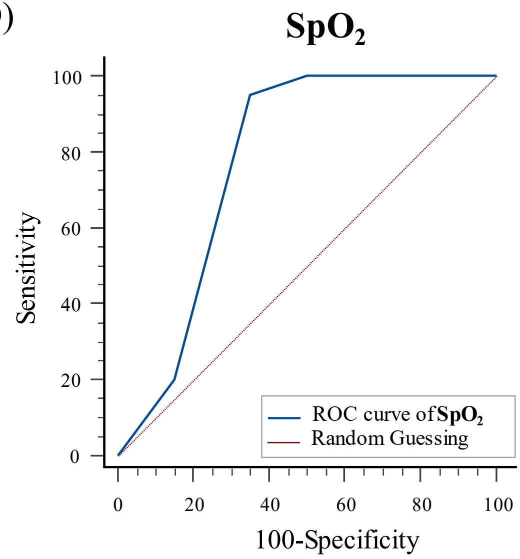

AUC	0.776
p-value	< 0.001
95% CI	0.617~0.893

Accuracy	80.00%
Recall	95.00%
F1 score	82.61%

SpO2(n=40)	Positive(P)	Negative(not P)
Shock	true positive=19	false negative=1
Non-Shock	false positive=7	true negative=13

Figure S1. Comparative Analysis of MAP, SBP, HR, and SpO_2 as Predictors of Shock: ROC Curves and Confusion Matrices (A) ROC curve of MAP: The analysis yielded an AUC of 0.833.

An AUC ranging from 0.7 to 0.9 indicates a moderate level of accuracy. The AUC of 0.833 exceeds the test standard, and the associated 95% CI was 0.681-0.982. The p value was less than 0.05, thus indicating statistical significance. This finding suggests that MAP is a significant predictor of shock. Confusion matrix for MAP: The criterion used to identify shock patients was MAP < 65 mmHg. This cutoff value had an accuracy of 85.00%, a recall of 70.00%, and an F1 score of 82.35%. **(B)** ROC curve of SBP: The analysis yielded an AUC of 0.929, indicating high accuracy for distinguishing shock patients from non-shock patients. The 95% CI was 0.801-0.986, and the p value was less than 0.01, indicating statistical significance. The model achieved an accuracy of 90.00%, indicating that 90% of the predictions were correct. The recall, or sensitivity, was 80.00%, indicating that 80% of actual shock cases were identified correctly, and the F1 score was 88.89%, indicating an effective balance of precision and recall. **(C)** ROC curve of HR: The analysis yielded an AUC of 0.947, indicating high accuracy for diagnosing shock. The 95% CI was 0.827 to 0.993, and the p value was less than 0.001, indicating statistical significance. The model achieved an accuracy of 90.00%, a recall of 90.00%, and an F1 score of 90.00%, indicating a good balance between precision and recall. **(D)** The ROC curve for SpO_2 demonstrated the diagnostic performance of this parameter in distinguishing shock patients from non-shock patients, with an AUC of 0.776, indicating moderate accuracy. The p value was less than 0.001, indicating statistical significance. SpO_2 achieved an accuracy of 80.00%, a recall of 95.00%, and an F1 score of 82.61%, thus demonstrating its effectiveness in identifying shock patients. These results suggest that SpO_2

is a reliable early diagnostic marker of shock, offering a balance between sensitivity and specificity, which is crucial for timely clinical intervention.

(A)

MAP Predicted

MAP(n=40)	Positive(P)	Negative(not P)
Shock	true positive=8	false negative=12
Non-Shock	false positive=0	true negative=20

Actual

Accuracy	70.00%
Recall	40.00%
F1 score	57.14%

(B)

SBP Predicted

SBP(n=40)	Positive(P)	Negative(not P)
Shock	true positive=11	false negative=9
Non-Shock	false positive=0	true negative=20

Actual

Accuracy	77.50%
Recall	55.00%
F1 score	70.97%

Figure S2. Confusion matrix of the use of the MAP and SBP to predict shock. (A) Confusion matrix for MAP: A cutoff value of MAP < 65 mmHg was used to differentiate shock patients (positive) from non-shock patients (negative)[S1]. For MAP, the accuracy was 70.00%, the recall was 40.00%, and the F1 score was 57.14%. **(B)** Confusion matrix for SBP: A cutoff value of SBP < 95 mmHg was used to differentiate shock patients from non-shock patients[S2]. For SBP, the accuracy was 77.50%, the recall was55.00%, and the F1 score was 70.97%.

Supplementary tables

Table S1. Basic information of the study group and the control group. Hypertension (H.T.N.), diabetes mellitus (D.M.), hyperlipidemia (H.L.D.), cardiovascular disease (C.V.D.), liver cirrhosis (L.C.), cerebral vascular accidents (C.V.A.), renal failure (R.F.), gastrointestinal diseases (GI disease), immune thrombocytopenia purpura (I.T.P.), and cancer (C.A.).

		**Study Group**		**Control Group**		*p* value	Cohen's *d*
		N=20		N=20			
		n(%); Mean ± SD	Median	n(%); Mean ± SD	Median		
Gender	Male	9(45)	-	9 (45)	-		
	Female	11(55)	-	11 (55)	-		
Shock type	Septic	10(50)	-	N/A	-		
	Neurogenic	1(5)	-	N/A	-		
	Cardiogenic	3(15)	-	N/A	-		
	Hypovolemic	6(30)	-	N/A	-		
Age (year)		67.1 ±16.1	66.5	65.3 ±15.9	66.5		0.1
Height (cm)		159.4 ±7.1	159.0	161.2 ±8.5	160.0		-0.2
Weight (kg)		59.7 ±13.5	61.0	67.9 ±14.5	67.0		-0.6
BP (mmHg)	MAP	71.7 ±12.3	69.2	99.9 ±12.6	101.7	<0.001	-2.3
	SBP	96.4 ±13.7	95.5	136.8 ±17.9	138.0	<0.001	-2.5
	DBP	59.4 ±13.4	59.0	81.4 ±11.7	84.5	<0.001	-1.7
BT (°C)		36.6 ±0.9	36.5	36.3 ±0.3	36.2		0.4
HR (bpm)		102.1 ±18.0	104.0	76.9 ±9.0	74.0	<0.001	1.8
RR (breathing/min)		20.6 ±2.2	20.0	18.3 ±0.9	18.0	<0.001	1.6
SpO_2 (%)		98.2 ±7.9	98.5	98.3 ±1.2	99.0		0.0
CRT(s)		4.3 ±2.0	4.0	2.3 ±0.6	2.0	<0.001	1.4
SI (HR/SBP)		1.1 ±0.2	1.1	0.6 ±0.1	0.5	<0.001	3.0
Lactate(mmol/L)		3.8 ±2.9	2.9	-	-	-	-
ROI_1		25959.8 ±10505.2	24363.5	37695.1 ±15519.5	36340.2	<0.05	-1.1
ROI_2		22543.2 ±10054.1	21083.1	29121.7 ±10120.4	27783.4	<0.05	-0.5
ROI Difference		5158.1 ±3123.8	3815.6	11080.8 ±9197.4	8454.3	<0.05	-0.6
Medical History	H.T.N.	12 (60)		9(45)			
	D.M.	10(50)		5(14)			
	H.L.D.	4(20)		1(5)			
	C.V.D.	10(50)		2(10)			
	L.C.	4(16)		0			
	C.V.A.	2(10)		1(5)			
	R.F.	4(20)		0			
	GI diseases	4(20)		1(5)			
	Epilepsy	1(5)		0			
	I.T.P.	1(5)		0			
	C.A.	6(30)		0			

Table S2. Basic information of the HRV parameters

	Study Group		Control Group		*p* value
	N=20		N=20		
	Mean±SD	Median	Mean±SD	Median	
Mean RR	750.60 ±34.1	744.0	741.6 ±61.1	722.6	<0.05
Median RR	724.1 ±46.3	717.5	709.9 ±85.6	705	
SDRR	165.7 ±43.0	168.5	141.6 ±60.6	161.3	
RMSSD	232.7 ±68.2	246.1	201.1 ±94.6	221.6	
SDSD	232.6 ±68.2	245.7	200.2 ±95.0	221.4	
SDRR_RMSSD	165.7 ±43.0	168.5	141.6 ±60.6	161.3	
HR	80.1 ±3.6	80.7	81.4 ±6.3	83.0	<0.05
PNN25	84.8 ±18.2	91.4	65.1 ±28.6	75.0	<0.001
PNN50	73.2 ±22.1	79.5	55.9 ±30.4	68.2	<0.05
SD1	164.5 ±48.2	173.8	141.6 ±67.2	156.5	
SD2	164.6 ±45.9	174.8	138.4 ±60.0	160.1	<0.05
KURT	-0.5 ±1.7	-0.9	1.9 ±5.2	-0.3	<0.001
VLF Power	24056.1 ±10682.6	24512.2	17969.8 ±14815.8	17950.8	<0.05
VLF PCT	1073.8 ±1076.5	725.6	1117 ±1439.1	581.0	
LF Power	6501.5 ±3944.4	6657.8	3960.4 ±4021.9	2529.4	<0.05
LF PCT	16480.8 ±8713.8	15594.2	12892.4 ±12105.6	10664.9	
LF NU	6.4 ±7.9	3.3	10.8 ±16.2	4.3	
HF Power	27.7 ±13.8	28.1	20.2 ±14.6	17.6	<0.05
HF PCT	65.9 ±15.3	67.8	69 ±20.1	71.1	
HF NU	29.7 ±15.3	28.5	23.2 ±15.6	20.2	
Tp	70.3 ±15.3	71.5	76.9 ±15.6	79.8	
LF Per HF	0.5 ±0.6	0.4	0.4 ±0.3	0.3	
HF Per LF	3.7 ±3.3	2.5	7.2 ±15.4	3.1	
SampEn	0.4 ±0.2	0.3	0.6 ±0.4	0.5	<0.001
HIGUCHI	1.4 ±0.1	1.4	0.7 ±2.8	1.4	

References

S1. J.-L. Vincent *et al.*, "Mean arterial pressure and mortality in patients with distributive shock: a retrospective analysis of the MIMIC-III database," *Annals of Intensive Care,* vol. 8, no. 1, p. 107, 2018.

S2. A. K. B. Kristensen, J. G. Holler, S. Mikkelsen, J. Hallas, and A. Lassen, "Systolic blood pressure and short-term mortality in the emergency department and prehospital setting: a hospital-based cohort study," *Critical Care,* vol. 19, no. 1, p. 158, 2015.
